# Bmi-1-induced miR-27a and miR-155 promote tumor metastasis and chemoresistance by targeting RKIP in gastric cancer

**DOI:** 10.1186/s12943-020-01229-y

**Published:** 2020-06-24

**Authors:** Yaqing Li, Zhenfeng Tian, Ying Tan, Guoda Lian, Shangxiang Chen, Shaojie Chen, Jiajia Li, Xuanna Li, Kaihong Huang, Yinting Chen

**Affiliations:** 1 Guangdong Provincial Key Laboratory of Malignant Tumor Epigenetics and Gene Regulation, Sun Yat-sen Memorial Hospital, Sun Yat-sen University, Guangzhou, 510120 P. R. China; 2 Department of Gastroenterology, Sun Yat-sen Memorial Hospital, Sun Yat-sen University, Guangzhou, 510120 P. R. China; 3 Department of Nephrology, Sun Yat-sen Memorial Hospital, Sun Yat-sen University, Guangzhou, 510120 P. R. China

**Keywords:** Bmi-1, RKIP, miR-27a, miR-155, Gastric cancer

## Abstract

**Background:**

We previously reported an inverse relationship between B cell-specific Moloney murine leukemia virus integration site 1 (Bmi-1) and Raf kinase inhibitory protein (RKIP), which is associated with the prognosis of gastric cancer (GC). In this study, we further explored the microRNA (miRNA) regulatory mechanism between Bmi-1 and RKIP.

**Methods:**

Microarray analysis was first carried out to identify miRNA profiles that were differentially expressed in cells overexpressing Bmi-1. Then, miRNAs that could regulate RKIP were identified. Quantitative real-time PCR (qRT-PCR) and Western blotting were performed to measure the expression of Bmi-1, miR-155, miR-27a and RKIP. RKIP was confirmed as a target of miR-27a and miR-155 through luciferase reporter assays, qRT-PCR and Western blotting. The effects of the Bmi-1/miR-27a/RKIP and Bmi-1/miR-155/RKIP axes on tumor growth, proliferation, migration, invasion, colony-formation ability, metastasis and chemoresistance were investigated both in vitro and in vivo.

**Results:**

The downregulation of RKIP by Bmi-1 occurred at the protein but not mRNA level. This indicates probable posttranscriptional regulation. miRNA expression profiles of cells with ectopic expression of Bmi-1 were analyzed and compared to those of control cells by microarray analysis. A total of 51 upregulated and 72 downregulated miRNAs were identified. Based on publicly available algorithms, miR-27a and miR-155 were predicted, selected and demonstrated to target RKIP. Bmi-1, miR-27a and miR-155 are elevated in human GC and associated with poor prognosis of GC, while RKIP is expressed at lower levels in GC and correlated with good prognosis. Then, in vitro tests shown that in addition to regulating RKIP expression via miR-27a and miR-155, Bmi-1 was also able to regulate the migration, invasion, proliferation, colony-formation ability and chemosensitivity of GC cells through the same pathway. Finally, the in vivo test showed similar results, whereby the knockdown of the Bmi-1 gene led to the inhibition of tumor growth, metastasis and chemoresistance through miR-27a and miR-155.

**Conclusions:**

Bmi-1 was proven to induce the expression of miR-27a and miR-155 and thus promote tumor metastasis and chemoresistance by targeting RKIP in GC. Overall, miR-27a and miR-155 might be promising targets for the screening, diagnosis, prognosis, treatment and disease monitoring of GC.

## Background

Gastric cancer (GC) is one of the leading causes of cancer morbidity and mortality worldwide, especially in Eastern Asian and Eastern European countries [1–3]. Patients in advanced stages benefit less than expected from palliative chemotherapies, mainly due to tumor metastasis and chemoresistance [4, 5], although the underlying molecular mechanism remains largely unknown. In our previous studies, the polycomb-group protein B cell-specific Moloney murine leukemia virus integration site 1 (Bmi-1) was reported to be associated with tumor size, clinical stage and prognosis of GC [6, 7]. Furthermore, we demonstrated that Bmi-1 is inversely associated with Raf kinase inhibitory protein (RKIP), a regulator of apoptosis induced by chemotherapeutic agents and a clinically relevant cancer metastasis suppressor gene [8, 9]. Based on previously reported results, the negative correlation between Bmi-1 and RKIP was deemed valuable in predicting patient survival and therapeutic response in GC [10]. The inverse expression pattern of Bmi-1 and RKIP was confirmed in GC cell lines by utilizing in vitro gene overexpression and silencing methods, suggesting the likelihood of RKIP being regulated by Bmi-1. However, the regulatory mechanism between Bmi-1 and RKIP needs further elucidation.

A variety of factors may be involved in the mechanism regulating the expression of Bmi-1 and RKIP. Among all potential regulatory mechanisms, microRNAs (miRNAs) emerged as a top research target due to their significance in various biological activities. miRNAs are an endogenous group of small noncoding RNAs that regulate up to 60% of human protein-coding genes at the posttranscriptional level by binding to the 3′ untranslated region (3’UTR) of a target mRNA [11–13]. miRNAs are able to affect tumor cell proliferation, invasion, metastasis and chemoresistance by regulating gene expression. For instance, Bmi-1 upregulates miR-21 and miR-34a in addition to regulating GC stem cell-like properties via the activation of the AKT-NF-κB pathway [14]. RKIP is also reported to be directly regulated by miR-543, which controls cell proliferation and metastasis in human prostate cancer cells [15]. Given the importance of miRNAs, the dysregulation of miRNAs is thought to be closely related to metastasis and chemoresistance in GC. We hypothesized that miRNAs may account for the negative correlation between Bmi-1 and RKIP. Therefore, this study aimed to explore Bmi-1-induced miRNAs that regulate RKIP. The candidate miRNAs were identified and predicted and were then validated by in vitro and in vivo experiments.

Overall, a novel microRNA regulatory mechanism of the Bmi-1-RKIP signaling axis was found. The inhibition of the RKIP tumor suppressor and a mechanism underlying tumor metastasis and resistance to chemotherapy in GC were also elucidated in this study. These findings may be significant in terms of screening, diagnosis, prognosis, disease monitoring and therapeutic value in GC.

## Methods

### Tissue specimens

Fifteen pathologically confirmed GC specimens with tumor and adjacent normal paired fresh-frozen tissues were obtained at Sun Yat-sen memorial hospital, Sun Yat-sen University (Guangdong, China). No preoperative treatments were received. Total RNA and protein of frozen tissues were extracted for quantitative real-time PCR (qRT-PCR) and Western blotting assays. The clinical specimens were obtained with patients’ informed consent and permission from the Institutional Ethics Committee of Sun Yat-sen memorial hospital. RNA-seq data, clinicopathological information and follow-up data of GC were downloaded from The Cancer Genome Atlas (TCGA) database (https://www.cancer.gov). Patients with censored overall survival time, gene expression profiles or certain pathological classification were excluded. The cut-off values of Bmi-1, miR-27a-3p (miR-27a), miR-155-5p (miR-155) and RKIP expression in Kaplan-Meier analysis were determined by X-tile software [16, 17].

### Cell lines

The human GC cell lines (BGC823 and SGC7901) were obtained from the Institute of Biochemistry and Cell Biology, Chinese Academy of Sciences (Shanghai, China). The human gastric epithelial immortalized GES-1 cell line was purchased from Beijing Institute for Cancer Research. The human leukemia monocytic U937 cell line was purchased from American Type Culture Collection (ATCC, Washington, USA). All these cell lines were cultured in Dulbecco’s modified Eagle’s medium (DMEM, Gibco, MA, USA) supplemented with 10% fetal bovine serum (FBS, Biological Industries, Beit Haemek, Israel) and incubated at 37 °C in a humidified atmosphere of 5% CO_2_.

### Plasmids, siRNAs and stable cell lines

pLNCX2-Bmi-1 was constructed as previously described [7]. Stable cells overexpressing Bmi-1, SGC7901-Bmi-1, BGC823-Bmi-1, GES-1-Bmi-1#1 and GES-1-Bmi-1#2 were generated by retroviral transfection as described in our previous study [7, 10]. The Bmi-1 overexpressing cells were also named Vector-Bmi-1, and the negative control was named Vector-Ctrl. Lentiviral shRNA virus specific to human Bmi-1 and Bmi-1-specific siRNAs were purchased from GenePharma (Shanghai, China). The shRNA sequences (the same as the specific siRNAs) for targeting Bmi-1 are shown in Supplementary Table S1. To construct cell lines for constitutive miRNA expression, lentiviruses containing GFP-miR-27a, GFP-miR-155, or GFP-negative control miRNA vector were purchased from GenePharma, Inc. The lentiviral vectors were used to infect targeted cells. BGC823 and SGC7901 cells were pre-seeded in a 6-well plate overnight and infected with 10 μl of virus. Infected cells were selected by adding 400 ng/ml puromycin for 5 days and then transferred to cultured flasks for proliferation. Stable cell lines were verified by qRT-PCR.

### Microarray data processing

The Bmi-1-overexpressing GES-1 cell line GES-1-Bmi-1#1 and its vector control cells were harvested with TRIzol, and miRNAs were extracted using a miRNeasy mini kit (QIAGEN, Beijing, China). Then, samples were sent to KangChen Bio-tech Inc. (Shanghai, China), quantified and analyzed the differential expression of miRNAs using a miRNA microarray. Microarray hybridization, data generation, and normalization were performed following standard protocols. Finally, differentially expressed miRNAs were identified through fold-change filtering. The cut-off fold change was 2.0.

### Luciferase assay

The RKIP 3’UTR was amplified by PCR separately using the primers described in Supplementary Table S1 from cDNA of SGC7901 cells. The PCR product was ligated into the multiple cloning region of the pGL3 luciferase reporter plasmid (Promega, Wisconsin, USA) according to the manufacturer’s recommendations. 293T cells plated in 96-well plates at a density of 4 × 10^4^ cells per well were cotransfected with 100 ng of the constructed luciferase plasmid or the control luciferase plasmid and 15 ng of the pRL-TK Renilla plasmid (Promega) using the Lipofectamine 2000 reagent (Thermo Fisher Scientific, MA, USA). miR-27a mimic/mut and miR-155 mimic/mut (50 nmol/L) were then cotransfected with the luciferase plasmid containing the RKIP 3’UTR for microRNA detection. After 24 h, cells were lysed and detected for Renilla and firefly luciferase activity using the Dual Luciferase Reporter Assay Kit (Promega). Three independent cotransfection experiments were carried out in triplicate.

### RNA electrophoretic mobility shift assays

All oligonucleotides and primers described as follows were obtained from GenePharma (Shanghai, China). The oligonucleotides hsa-miR-27a: 5′-UUCACAGUGGCUAAGUUCCGC-3′ and hsa-miR-155: 5′- UUAAUGCUAAUCGUGAUAGGGGU-3′ were synthesized and 5′-labeled with Cy5 dye. The 2′-O-methyl-modified RNA oligonucleotides RKIP-miR-27a: 5′-GGGGUAUUUUGGUACUGUGAU-3′ and RKIP-miR-155: 5′-AGUUGCUGAAUGUUGCAUUAAU-3′, which correspond to the hsa-miR-27a and hsa-miR-155 targeting sequences present in the 3’UTR of RKIP, were 5′-labeled with FAM dye. Cytoplasmic extracts of SGC7901 cells were prepared using NEPER Nuclear and Cytoplasmic extraction reagents (Thermo Scientific, MA, USA). The RNA EMSA was carried out according to the manufacturer’s instructions (Thermo Fisher Scientific) and our previous studies [18, 19].

### Animal experiments

All mouse experiments were conducted in accordance with the Guide for the Care and Use of Laboratory Animals by the National Institutes of Health and were approved by the Animal Care and Use Committee of Sun Yat-sen University. To study the effect of the Bmi-1/miR-27a/RKIP and Bmi-1/miR-155/RKIP axes on GC tumorigenesis, chemoresistance and metastasis, we conducted tumor xenograft and tail vein injection studies in mice.

For the tumorigenesis experiments, BGC823-shcon, BGC823-shBmi-1, BGC823-NC mimic, BGC823-miR-27a mimic, BGC823-miR-155 mimic, BGC823-shBmi-1 + NC mimic, BGC823-shBmi-1 + miR-27a mimic and BGC823-shBmi-1 + miR-155 mimic cells (1.5 × 10^6^ cells in 0.15 ml of PBS) were subcutaneously inoculated into the right flanks of female BALB/c nude mice (5 weeks old) to establish tumor xenografts. Tumor size was monitored and calculated every 3 days. The tumor volume was estimated using the equation: tumor volume (mm^3^) = (length in mm) × (width in mm)^2^/2. In the chemoresistance experiments, tumor-bearing xenografts were established the same as in the tumorigenesis experiment. Once the subcutaneous tumors grew big enough to be palpable, intraperitoneal (IP) injection of either 5-Fu (15 mg/kg) or vehicle control (PBS) was performed every other day for 14 days. On the 21st day after implantation, mice in the tumorigenesis and chemoresistance groups were sacrificed. For the tumor metastasis experiment, 1 × 10^6^ cells were injected into mice via the tail vein. The mice were sacrificed 30 days after cell administration. The tumors, lungs or livers dissected in all animal experiments were fixed and paraffin-embedded for histopathological analysis.

### Immunohistochemistry

Paraffin-embedded tissue sections that were 4 μm thick were used in immunohistochemistry. Following deparaffinization, hydration, antigen retrieval and blocking, the slides were successively incubated with primary antibodies at 4 °C overnight and secondary antibodies conjugated with horseradish peroxidase (HRP) at 37 °C for 30 min. The slides were then incubated in DAB solution for 10 min and counterstained with hematoxylin for 1 min. Images of the stained slides were obtained with a microscope (Nikon, Tokyo, Japan).

### Statistical analysis

All statistical analyses were performed with Statistical Product and Service Solutions version 22.0 (SPSS, Illinois, USA) and GraphPad Prism 5.0 software (GraphPad, Inc., CA, USA). Data are presented as the mean ± standard error (^−^x ± SE). The statistical significance of the differences was determined by t-test and one-way ANOVA test according to the homogeneity of variances. *P* < 0.05 was considered to be statistically significant.

The detailed methodology of qRT-PCR, Western bloting and functional experiments can be found in the Supplementary Materials.

## Results

### microRNAs induced by Bmi-1 that regulate RKIP expression were screened and selected

**Fig. 1:**
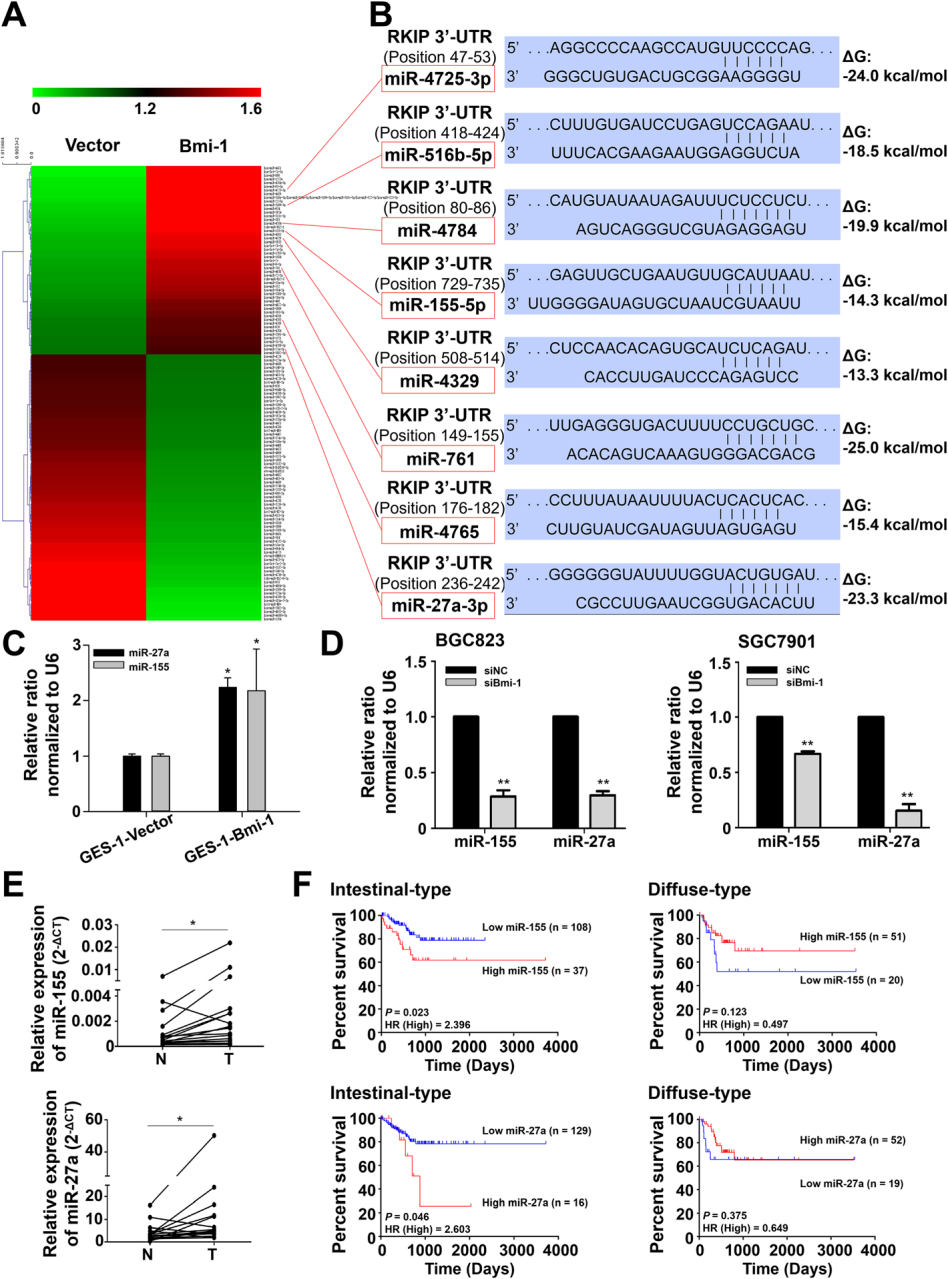
Candidate microRNAs that may be influenced by Bmi-1 expression. **a** Hierarchical clustering of microarray data showing the comparison of miRNA expression profiles between the two groups (GES-1-Bmi-1 cells and their control). **b** RKIP was predicted as a target of these differentially upregulated miRNAs, and their predicted binding site in the RKIP 3’UTR is shown. **c** qRT-PCR analysis of differentially expressed miR-27a and miR-155 in GES-Bmi-1 cells. *P < 0.05 vs. GES-1-Vector. **d** The gene expression levels of miR-155 and miR-27a were downregulated by siBmi-1 GC cells. **P < 0.01 vs. siNC. **e** qRT-PCR analysis of miR-27a and miR-155 expression in 15 paired GC tissues (T) and adjacent normal tissue samples (N). *P < 0.05. **f** Kaplan-Meier showed the correlations between miR-155 and miR-27a and the 3-year overall survival of patients with intestinal-type or diffuse-type GC from TCGA

miRNAs have been confirmed to function in disease pathogenesis by regulating the expression of genes mainly in posttranscriptional gene silencing. To determine whether miRNAs are involved in Bmi-1 repression of RKIP, we compared the miRNA expression profiles of cells with ectopic expression of Bmi-1 (GES-1-Bmi-1) with the control cells (GES-1). Microarray analysis identified 51 upregulated and 72 downregulated miRNAs in GES-1-Bmi-1 cells (Fig. 1a and Supplementary Table S2), including miR-27a, miR-155, and miR-516, which were upregulated and had been previously found to be associated with gastric adenocarcinoma or other gastrointestinal tumor invasiveness. Among all the differentially upregulated miRNAs, miR-27a, miR-761, miR-155, miR-4725, miR-4784, miR-4329, miR-4765 and miR-516b were predicted to target RKIP based on analysis with publicly available algorithms (Fig. 1b). miR-27a and miR-155 were selected and proven to be upregulated in GES-Bmi-1 cells by qRT-PCR (Fig. 1c). As shown in Fig. 1d, miR-27a and miR-155 were downregulated when the Bmi-1 gene was knocked down in BGC823 and SGC7901 GC cell lines.

### The clinical significance of Bmi-1, miR-27a, miR-155 and RKIP

The association between Bmi-1, RKIP and the clinical outcome of gastric cancer has been reported in our previous study [10]. To further explore the clinical significance of Bmi-1, miR-27a, miR-155 and RKIP in GC, we first analyzed their RNA expression levels by qRT-PCR. The results demonstrated higher Bmi-1, miR-27a and miR-155 expression and lower RKIP expression in GC tissues (Fig. 1e and Fig. S1A). Furthermore, Western blotting examination revealed that 93.33% (14/15) of patients with GC had an upregulated protein level of Bmi-1, and 73.33% (11/15) had a downregulated protein level of RKIP (Fig. S1B). The inverse association between Bmi-1 and RKIP is consistent with our previous research results. Moreover, we analyzed the RNA expression levels of Bmi-1, miR-27a, miR-155 and RKIP in human GC samples from TCGA database using StarBase (http://starbase.sysu.edu.cn) [20]. As shown in Fig. S1D, Bmi-1, miR-27a, and miR-155 were significantly upregulated, while RKIP was downregulated in GC, which is consistent with Fig. 1e and Fig. S1A. We divided the patients with available histological information from TCGA into two groups by Lauren classification (intestinal-type and diffuse-type) [21]. Tumors exhibiting signet ring type were classified as diffuse. Patients with high expression of Bmi-1, miR-27a, and miR-155 and low expression of RKIP had a shorter 3-year overall survival in intestinal-type GC. Moreover, there was no significant correlation between the above four indicators and the 3-year overall survival in diffuse-type GC (Fig. 1f and Fig. S1C). The above results suggest that these four indicators may have different clinical significance in different pathological classifications of GC. Taken together, our results reveal that Bmi-1, miR-27a and miR-155 are elevated in human GC and associated with poor prognosis of GC, while RKIP is expressed at lower levels in GC and correlated with good prognosis.

### miR-27a and miR-155 regulate RKIP directly

**Fig. 2:**
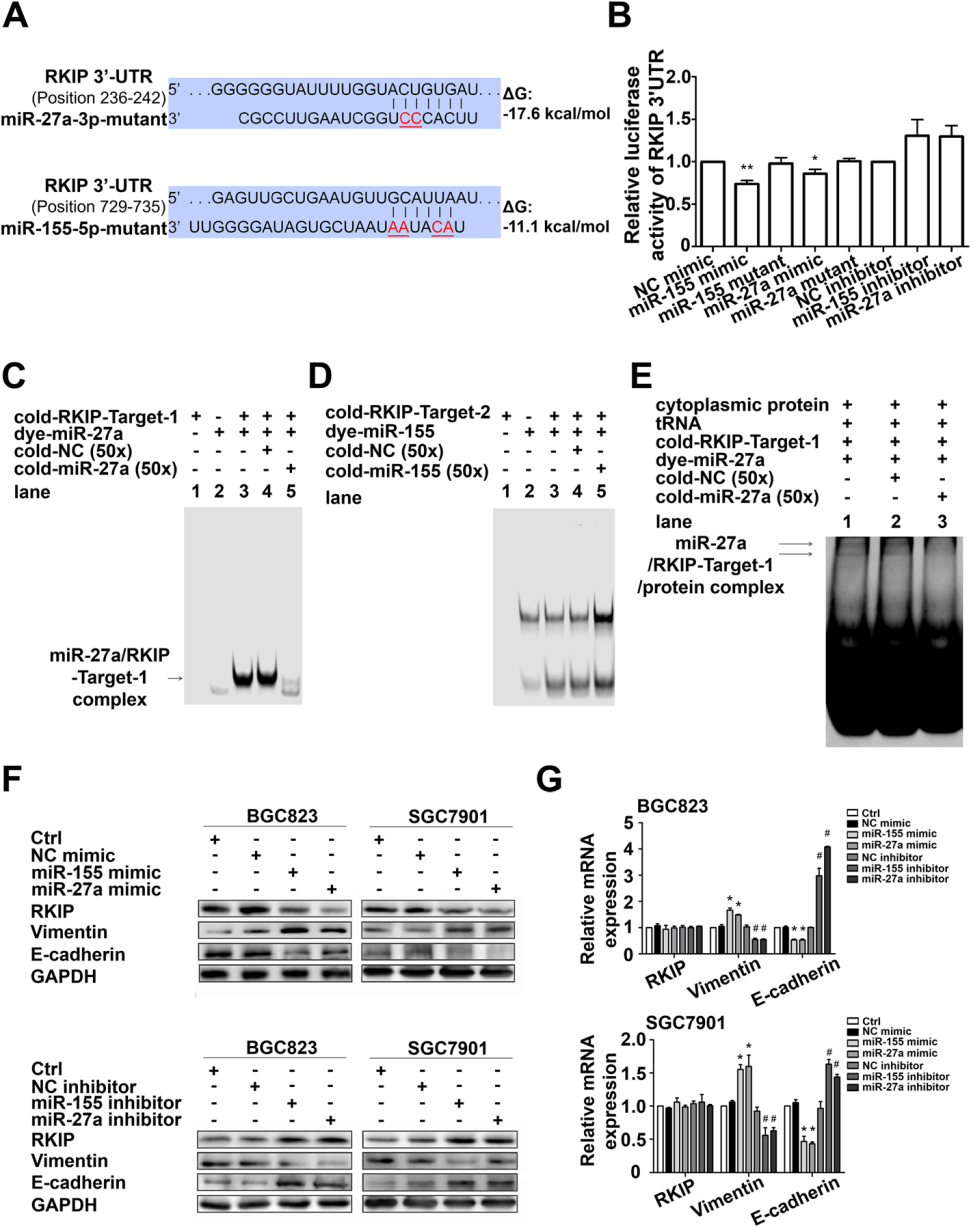
miR-155 and miR-27a target RKIP, negatively regulate its expression, and promote the epithelial-mesenchymal transition (EMT) process. **a** The predicted binding site on the RKIP 3’UTR for miR-155 and miR-27a and the mutant sequence. **b** The relative luciferase activity of 293 T cells cotransfected with miRNAs and the indicated luciferase plasmid. *P < 0.05 vs. NC mimic, **P < 0.01 vs. NC mimic. **c** EMSA showed the formation of a stable dye-miR-27a/RKIP-Target complex, which was inhibited by the competitive interaction between excessive amounts of cold-miR-27a (unlabeled miR-27a) and RKIP-Target. **d** EMSA demonstrated no interaction between miR-155 and RKIP-Target. **e** The formation of a miR-27a/RKIP-Target protein complex by adding a cell cytoplasmic extract. **f** Western blot analysis showed the expression of RKIP and EMT-related marker genes after treatment with miR-155 mimic/inhibitor or miR-27a mimic/inhibitor. **g** Gene expression of RKIP and EMT-related marker genes modulated by miR-155 mimic/inhibitor or miR-27a mimic/inhibitor. *P < 0.05 vs. NC mimic, ^#^*P* < 0.05 vs. NC inhibitor

As shown in Fig. 2a and b, miR-27a and miR-155 significantly decreased the luciferase activity of the constructed plasmid containing the 3’UTR of RKIP. In addition, RNA EMSAs were performed to demonstrate the direct interaction between miR-27a and its cognate RKIP target sequence, but such interaction was not observed in miR-155. As shown in Fig. 2c, miR-27a and its cognate RKIP mRNA oligonucleotide could bind together to form an electrophoretically stable miRNA/mRNA complex. However, miR-155 displayed two bands at different migration rates by PAGE, indicating that it did not bind with the oligonucleotide of RKIP mRNA to form a stable complex (Fig. 2d). Furthermore, the miR-27a/RKIP mRNA complex was able to interact with SGC7901 cytoplasmic extracts to form new complexes (Fig. 2e, lane 1). To address the role of miR-27a and miR-155 in RKIP expression, Western blot analysis and corresponding densitometry analysis were performed in BGC823 and SGC7901 GC cell lines after transfection with either miR-27a mimic/inhibitor or miR-155 mimic/inhibitor. The results showed that RKIP protein expression was reduced in the miR-27a and miR-155 mimic groups but increased in the miR-27a and miR-155 inhibitor groups compared to the control groups (Fig. 2f and Fig. S3A). However, the RNA expression of RKIP did not change after transfection with the miRNAs (Fig. 2g).

### Bmi-1 regulates RKIP expression through miR-27a and miR-155 in GC cells

**Fig. 3:**
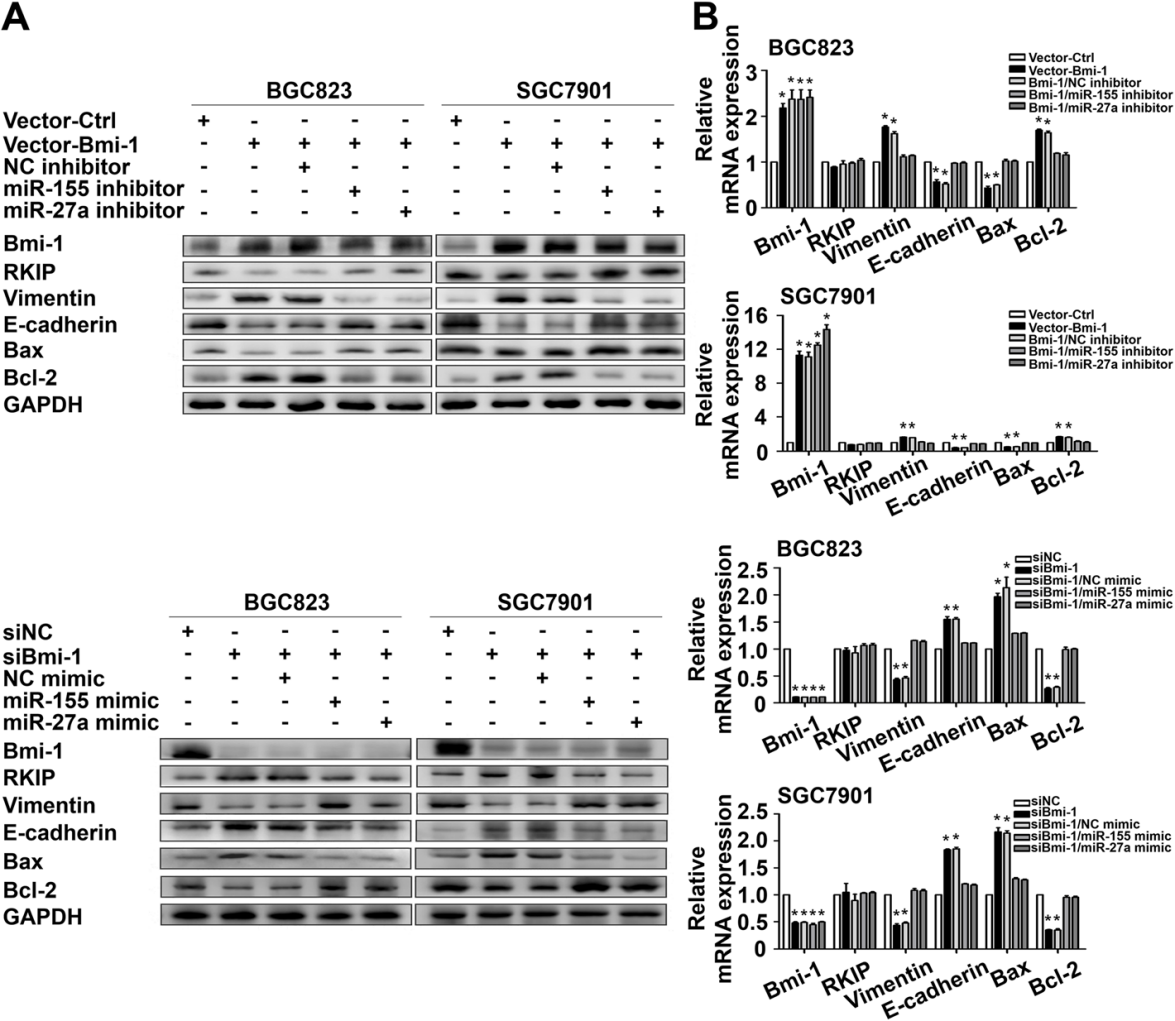
Bmi-1 regulates RKIP expression through miR-27a and miR-155 in GC cells. **a** The inhibition of miR-27a and miR-155 weakened the influence of Bmi-1 overexpression on RKIP downregulation, whereas the increased expression of RKIP after transfection of shBmi-1 was attenuated by miR-155 or miR-27a. **b** The results of qRT-PCR were in accordance with the Western blotting assays. *P < 0.05 vs. Vector-Ctrl/siNC

To confirm that the regulatory effects of Bmi-1 on RKIP are mediated through miR-27a and miR-155, we introduced miR-27a inhibitor and miR-155 inhibitor to GC cells with ectopic expression of Bmi-1 and miR-27a mimic and miR-155 mimic to GC cells with Bmi-1 gene knockdown. Although the protein expression of RKIP in BGC823 and SGC7901 cells decreased considerably after transfection with Bmi-1, both the miR-27a and miR-155 inhibitors were able to restore RKIP expression. In contrast, the protein expression of RKIP in BGC823 and SGC7901 cells substantially increased after transient transfection with siBmi-1, and the miR-27a mimic and miR-155 mimic repressed RKIP expression (Fig. 3a and Fig. S3B). Besides, the RNA expression of RKIP did not change after transfection with the miRNAs or siBmi-1 (Fig. 3b). Altogether, these findings suggest that RKIP expression is negatively regulated by Bmi-1 through miR-27a and miR-155 in GC cell lines.

### Bmi-1 regulates cell migration, invasion, proliferation, colony-formation ability and chemosensitivity via miR-27a and miR-155 in vitro

To explore the miR-27a- and miR-155-mediated regulatory effects of Bmi-1 on RKIP in terms of GC progression, we infected BGC823 and SGC7901 cells with lentiviruses carrying miR-155 mimic, miR-27a mimic, shBmi-1, shBmi-1 + miR-27a mimic and shBmi-1 + miR-155 mimic. Stably transfected cells were established after 5 days of puromycin selection.

**Fig. 4:**
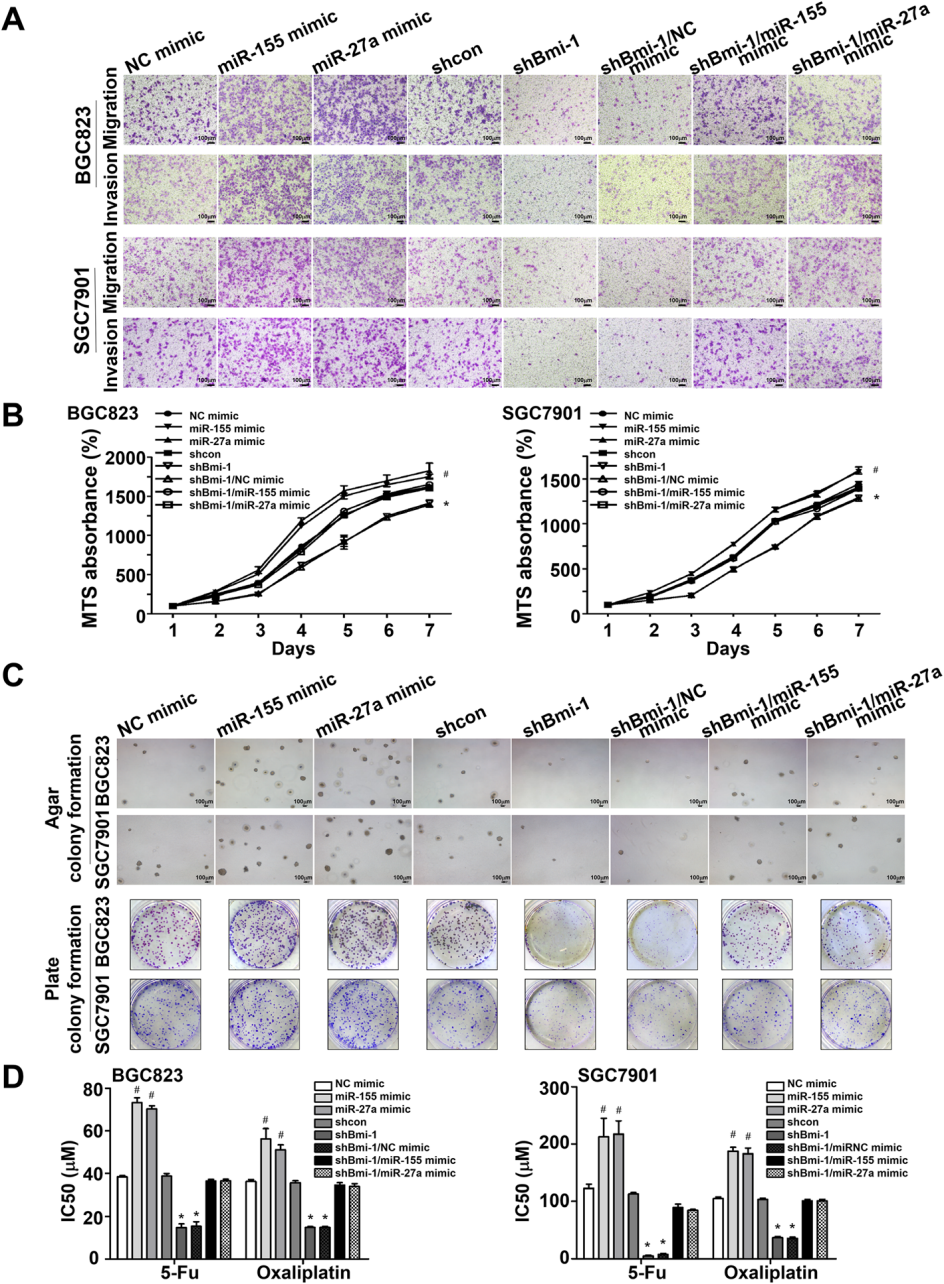
Bmi-1 regulates cell migration, invasion, proliferation and chemosensitivity via miR-155 and miR-27a in vitro. **a** Bmi-1 knockdown inhibited cell migration and invasion, which were enhanced by miR-155 or miR-27a (100 × magnification). **b** miR-155 or miR-27a improved cell proliferation, which was inhibited by Bmi-1 suppression. **c** Colony formation assays either in soft agar or on plates demonstrated that the shBmi-1 group generated fewer colonies than any other group, and the effect could be reversed by miR-155 or miR-27a. **d** MTS was performed to evaluate the IC_50_ values of cells upon different treatments. The inhibition of Bmi-1 enhanced chemosensitivity, while miR-155 and miR-27a increased the IC_50_. *P < 0.05 vs. shcon, ^#^*P* < 0.05 vs. NC mimic

The migratory and invasive behaviors of BGC823 and SGC7901 cells were detected using Transwell assays. We found that Bmi-1 gene knockdown reduced the number of migrating and invading cells compared to the mock control, whereas cotransfection of miR-27a or miR-155 increased the number of such cells (Fig. 4a and Fig. S3C).

The MTS experiments indicated that the overexpression of miR-27a and miR-155 led to an increase in cell proliferation, while the downregulation of Bmi-1 inhibited cell proliferation, which could be counteracted by cotransfection of miR-27a or miR-155 (Fig. 4b). Colony formation assays both in soft agar and culture plates demonstrated that compared to the negative control, fewer colonies developed when Bmi-1 was inhibited. However, cotransfection of miR-27a or miR-155 reversed the reduced colony-formation abilities (Fig. 4c).

Chemotherapy sensitivity of the tumor cells was then assessed using the MTS assay, and it was observed that Bmi-1-knockdown cells displayed less chemoresistance than control cells when treated with two conventional chemotherapeutic agents (5-Fu and oxaliplatin). Moreover, to determine whether Bmi-1 plays an important role in apoptosis via miR-27a or miR-155 during drug treatment, the miR-27a mimic and miR-155 mimic were also transfected into shBmi-1 cells. We found that the miR-27a mimic and miR-155 mimic significantly attenuated the impact of Bmi-1 silencing on drug-induced apoptosis (Fig. 4d). Moreover, the miR-27a inhibitor and miR-155 inhibitor could also weaken the effects of Bmi-1 overexpression on migration, invasion and chemoresistance in gastric cancer cells (Fig. S4 and Fig. S3C). Therefore, it can be deduced that Bmi-1 partly regulates chemosensitivity via a microRNA-dependent mechanism.

### Bmi-1 knockdown inhibits tumor growth, metastasis and chemoresistance via miR-27a and miR-155 in vivo

To demonstrate the functions of Bmi-1 and miR-27a/miR-155 in tumor growth, metastatic colonization and drug resistance, BGC823-NC mimic, BGC823-miR-155 mimic, BGC823-miR-27a mimic, BGC823-shcon, BGC823-shBmi-1, BGC823-shcon+NC mimic, BGC823-shBmi-1 + miR-155 mimic and BGC823-shBmi-1 + miR-27a mimic cells were injected subcutaneously or intravenously into nude mice.

**Fig. 5:**
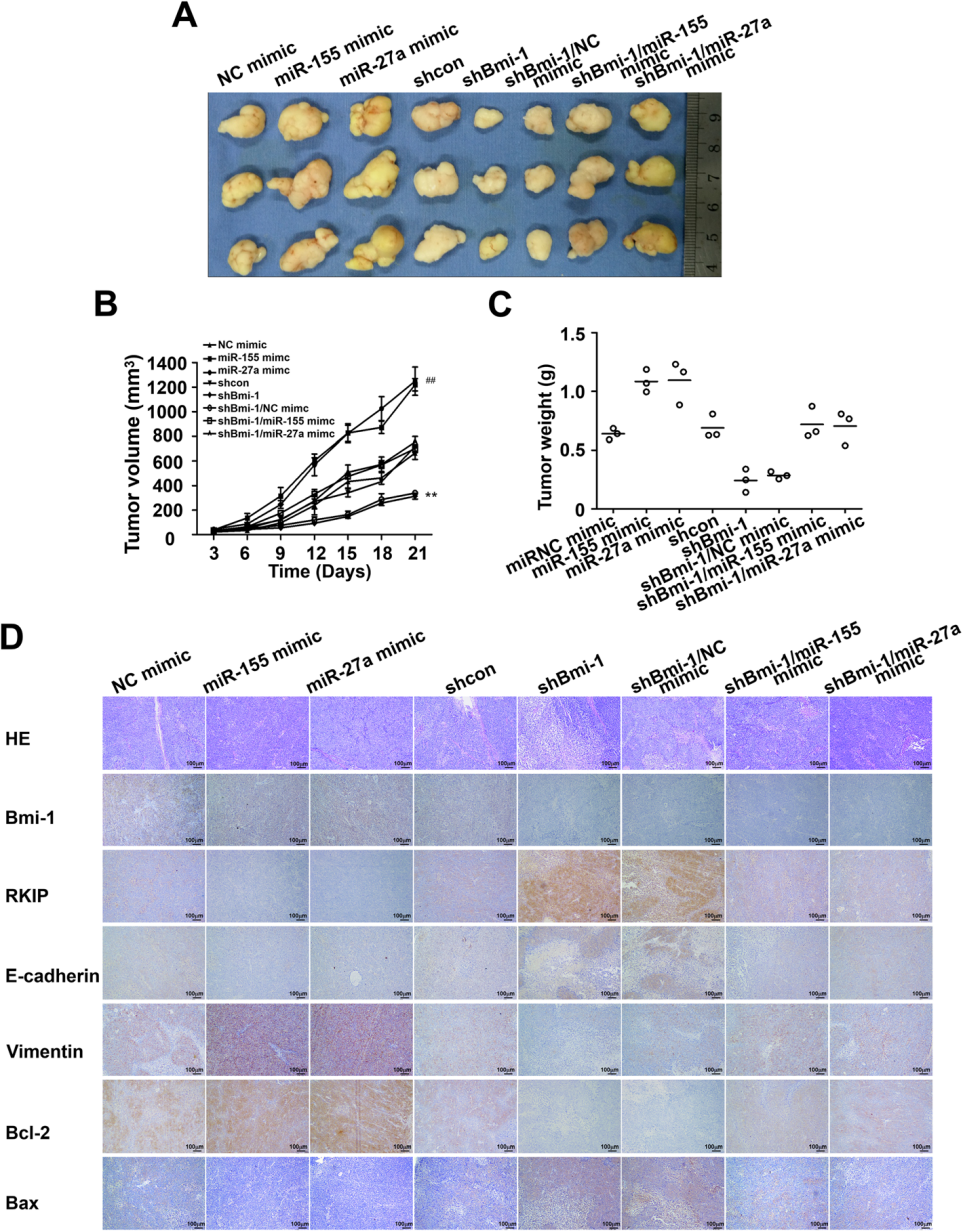
Bmi-1 knockdown inhibits tumor growth via miR-27a and miR-155 in vivo. **a** Image of tumors excised from xenograft models. **b** Tumor growth curve. **P < 0.01 vs. shcon, ^##^*P* < 0.01 vs. NC mimic. **c** Weight of the tumor harvested at the study endpoint. **d** Immunohistochemistry of tumors for the detection of Bmi-1, RKIP, Bcl-2, Bax and EMT-associated marker genes (100 × magnification)

**Fig. 6:**
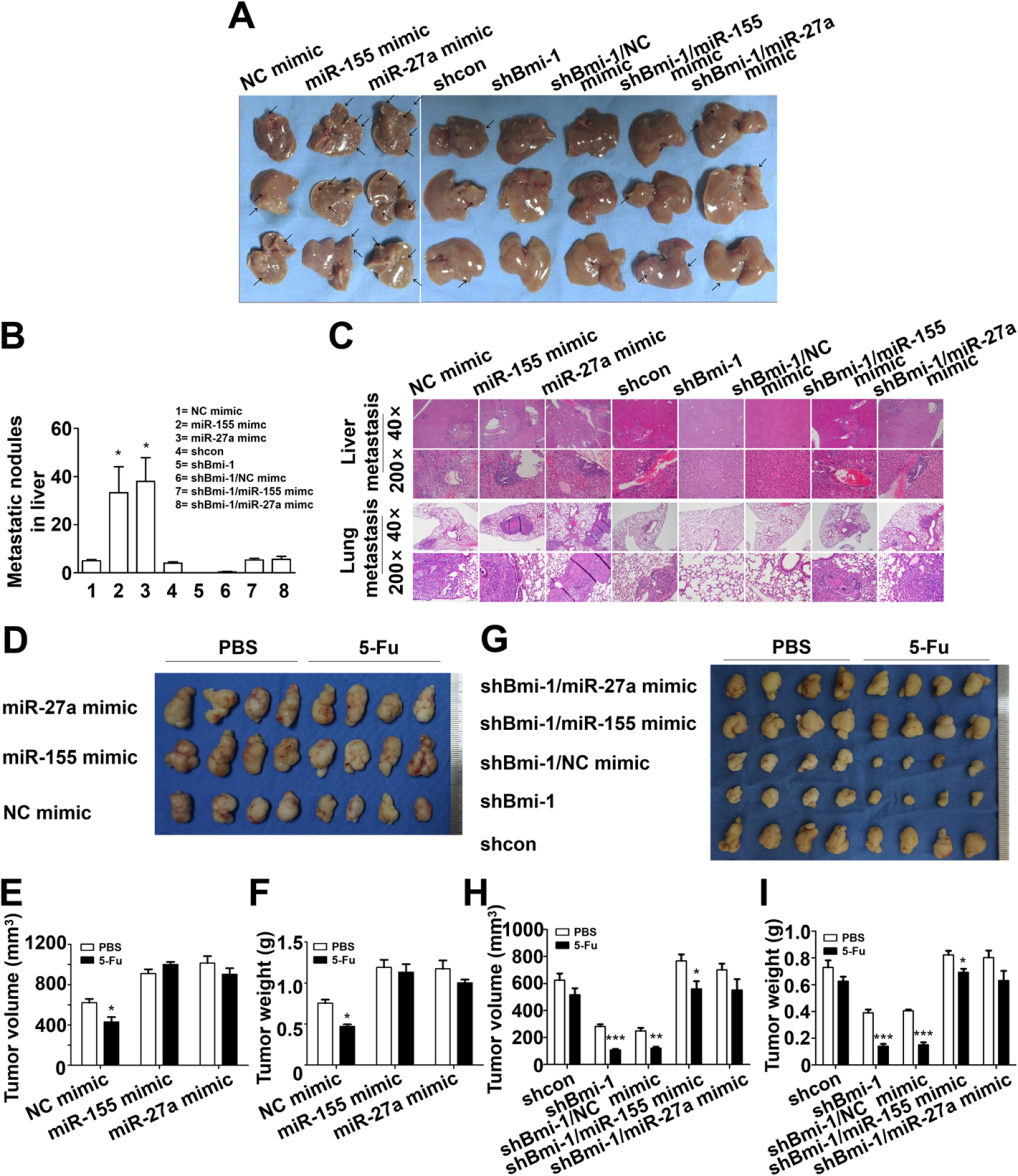
miR-155 and miR-27a lead to cancer metastasis and chemoresistance and attenuate the effect of shBmi-1 in vivo. **a** and **b** Imaging and quantification of metastatic nodules in liver specimens from metastatic models stablished by tail vein injection of BGC823 cells. Arrows show metastatic nodules. *P < 0.05 vs. NC mimic. **c** Hematoxylin and eosin (H&E) staining of BGC823 liver metastatic tumors. **d** and **g** Isolated tumors of xenograft models with intraperitoneal injections of PBS or 5-Fu. **e** and **f** The size and weight of subcutaneous tumors that were established by cells with stable overexpression of miR-155 or miR-27a and treated with 5-Fu. *P < 0.05 vs. PBS. **h** and **i** The size and weight of excised tumors from mice subcutaneously implanted with stably transfected cells. *P < 0.05 vs. PBS, **P < 0.01 vs. PBS, ***P < 0.001 vs. PBS

For the indicated time after subcutaneous injection, an obvious attenuation in tumor growth was observed in mice that were injected with shBmi-1, whereas mice that were injected with the miR-155 mimic or miR-27a mimic showed the opposite effect and had increased tumor growth (Fig. 5a-c). This was also confirmed by the immunohistochemical results (Fig. 5d). As expected, we observed that the BGC823-shBmi-1 group had fewer lung and liver metastases than the controls. We also noted that the BGC823-miR-27a mimic and BGC823-miR-155 mimic groups exhibited a significant increase in the number of metastatic foci in both the lungs and livers. Moreover, a dramatic upregulation was found in the number of foci in the lungs and livers of mice injected with BGC823-shBmi-1 + miR-155 mimic or BGC823-shBmi-1 + miR-27a mimic cells relative to BGC823-shBmi-1 (Fig. 6a-c).

To assess the chemosensitivity of tumor cells, we established subcutaneous xenografts and then evaluated their response to 5-Fu treatment. After 14 days of treatment, the mice were sacrificed, and their tumors were resected and weighed. The results demonstrated that the tumors were significantly larger in the miR-155 mimic and miR-27a mimic groups compared to the control group regardless of what therapy the animals had received, showing tolerance to 5-Fu (Fig. 6d-f). However, shBmi-1 displayed a significantly stronger effect in reducing tumor growth and increasing the sensitivity towards 5-Fu compared to the control. Notably, tumors in the groups cotransfected with shBmi-1 + miR-155 mimic or shBmi-1 + miR-27a mimic grew much faster than tumors in the shBmi-1 group, indicating the ability of the miR-155 mimic and miR-27a mimic to decrease the chemosensitivity of BGC823 cells to 5-Fu (Fig. 6g-i). Overall, the effect of shBmi-1 could be attenuated by reducing RKIP expression through the action of the miR-27a mimic or miR-155 mimic. Additionally, the results of immunohistochemical staining of Bmi-1, RKIP, Vimentin, BCL2 Associated X, Apoptosis Regulator (Bax) and B-cell lymphoma-2 (Bcl-2) detected in isolated tumors (Fig. S5A and B) were in accordance with the results of the Western blot assay. Moreover, the negative correlation between the expression of Bmi-1 and RKIP was further proven by microscopic observation of tumors derived from the shcon and shBmi-1 xenograft models, which provided further evidence to support our hypothesis. Therefore, based on these indications from correlation studies, it can be concluded that the Bmi-1/miR-27a/RKIP and Bmi-1/miR-155/RKIP axes are major determinants of cell growth, metastasis and resistance to chemotherapy in GC.

## Discussion

Recently, therapies targeting metastasis and chemoresistance of GC have attracted the attention of researchers [22, 23]. However, insufficient understanding of the underlying molecular mechanisms involved creates a barrier for the development of effective strategies in clinical practice. We previously reported the distinctive biological activity of Bmi-1/RKIP and their inverse relationship in GC, but the detailed mechanisms needed further investigation. In this study, we screened for Bmi-1-induced miRNAs and successfully identified the Bmi-1/miR-27a/RKIP and Bmi-1/miR-155/RKIP signaling axes. For the first time, we elucidated the molecular mechanism of how Bmi-1 regulates RKIP in GC, which ultimately affects the metastasis and chemoresistance of GC. Our results suggest that therapies targeting the Bmi-1/miR-27a/RKIP and Bmi-1/miR-155/RKIP signaling axes would be effective in GC patients, especially those with a high risk of metastasis and chemoresistance.

RKIP is a proven metastasis suppressor protein in various human cancer types and is linked to an invasive phenotype [24, 25]. Low levels or the absence of RKIP expression in GC is associated with poor patient prognosis [10, 26]. However, the molecular mechanisms of how RKIP expression is downregulated in GC remain unknown. Polycomb group (PcG) proteins are epigenetic gene-silencing proteins that have been shown to play important roles in human cancer occurrence and progression [27, 28]. Bmi-1, the first functionally identified PcG member, is frequently dysregulated in various cancers and strongly correlates with tumor aggressiveness; thus, its presence predicts a poor prognosis [10, 29, 30], but little attention has been paid to the downstream regulatory mechanism of Bmi-1 in GC promotion. The opposite biological functions and negative correlation between Bmi-1 and RKIP suggested a potential regulatory mechanism in GC. The results from our previous study led to the likely hypothesis that RKIP is regulated by Bmi-1 [10].

To directly examine the role of Bmi-1 in RKIP expression, we introduced a Bmi-1 expression plasmid by retroviral infection into the nonmalignant human gastric epithelial GES-1 cell line. RKIP protein expression was suppressed by ectopic expression of Bmi-1 in GES-1 cells [10]. However, forced expression of Bmi-1 barely changed the mRNA level of RKIP (Fig. S2A). Therefore, RKIP protein expression is likely to be regulated at the posttranscriptional level in GC cells. This result is in line with previous reports that have suggested the critical role of posttranscriptional mechanisms in the regulation of RKIP expression in human hepatocellular carcinoma [31]. Mechanisms such as protein degradation and microRNA-mediated suppression may also play a role in the posttranscriptional regulation of the expression of specific genes. Moreover, protein half-life analysis demonstrated that forced expression of Bmi-1 did not induce RKIP protein degradation (Fig. S2B). On the other hand, microRNAs are RNAs with sizes between 20 and 25 nucleotides, and they contribute to gastric carcinogenesis by regulating the expression of oncogenes and tumor suppressors at the posttranscriptional level to affect cell proliferation, apoptosis, motility and invasion [32–34]. microRNAs have been shown to be involved in regulating RKIP expression in human cancer [24, 35]. To our knowledge, this study is the first to report a difference in miRNA expression based on the overexpression of Bmi-1 in GES-1 cells. Potential miRNAs targeting RKIP were selected using miRNA microarray and publicly available algorithms (TargetScan). Upon combining the outcomes of the literature review, miR-27a and miR-155 were identified as candidate microRNAs that are involved in the Bmi-1 repression of RKIP.

In this study, we demonstrated that Bmi-1 functions as a repressor of RKIP, which is mediated by miR-27a and miR-155. miR-27a and miR-155 were also able to directly repress RKIP expression, which consequently affected the expression of genes necessary for regulating metastasis and chemoresistance, including Vimentin, Cadherin 1 (E-cadherin), Bax and Bcl-2. Through in vitro and in vivo experiments, it was observed that Bmi-1-downregulated cells showed slower growth, poorer migration and invasion capacities, and a clear sensitivity to in vitro drug treatment. Further in vivo experiments in mice demonstrated that miR-27a and miR-155 downregulated RKIP and promoted metastasis to the lungs and livers. The level of inhibition to oxaliplatin and 5-Fu sensitivity was also greater than that of the control. Furthermore, the miR-27a mimic and miR-155 mimic could significantly counteract the metastasis and chemoresistance effects in Bmi-1-downregulated cells. Similarly, the miR-27a inhibitor and miR-155 inhibitor weakened the effects of Bmi-1 overexpression on migration, invasion and drug resistance in gastric cancer cells. The results were consistent with previous results that highlight the significant role of RKIP in altering tumor cell metastasis and chemoresistance both in vitro and in vivo. In addition, we observed differences in miR-27a mimic- and miR-155 mimic-induced cell proliferation and the size of the tumors when compared to control cells. This implies that there may be other pathways influencing cancer growth simultaneously. Collectively, these findings strongly indicate the pivotal role of RKIP in Bmi-1-mediated promotion of metastasis and suppression of chemosensitivity.

Some researchers have reported that miR-27a is highly expressed in GC tissues and that the overexpression of miR-27a promotes the tumorigenicity, metastasis and chemoresistance of GC [36–38], which is in accordance with this study. In addition, previous studies illustrated that increased expression of miR-155 was closely related to tumor invasion and metastasis in advanced GC, implying poor prognosis [39]. Qu Y et al. showed that miR-155 downregulated the expression of TGFβR2 to promote the proliferation and migration of GC cells, which is consistent with our study [40]. Moreover, it was reported that *Helicobacter pylori* infection was closely linked to miR-155, possibly upregulating miR-155 expression to inhibit the DNA mismatch repair (MMR) gene and induce a mutant phenotype that is conducive to error-prone translation synthesis and thus promotes GC progression [41–43]. However, some studies have reported that miR-155 is expressed at low levels in GC tissue and acts as a tumor suppressor gene [44–46]. Combined with the prognostic analysis of miR-155 shown in Fig. 1f, we propose that miR-155 plays different roles in GC of different histological types. Therefore, the occurrence and development of GC is complex. Although the carcinogenic role of miR-27a and miR-155 in GC has been reported, our study demonstrates that miRNA as a key junction plays a posttranscriptional regulatory role in the Bmi-1/RKIP pathway, further revealing the specific molecular mechanism of GC metastasis and chemoresistance.

Previous published literature illustrates that GC is histologically complex and can be characterized by the expression profile of microRNAs. It was reported that miR-105, miR-145, and miR-133a were upregulated in diffuse-type lesions, while miR-498 and miR-494 were upregulated in intestinal-type GC [47, 48]. We analyzed the clinical significance of miR-27a and miR-155 from TCGA and found that these two indicators were not identical in different histological types, suggesting that these two indicators could be signatures linked to the tumorigenesis and development of GC. Therefore, we need to include a larger patient population and collect follow-up information to clarify the correlation between miR-27a, miR-155 and clinical prognosis in further studies. Moreover, we will verify the expression of miR-27a and miR-155 and its clinical significance in different histological types.

## Conclusions

In conclusion, the present study indicates that Bmi-1 negatively regulates the metastasis suppressor gene RKIP via microRNA-mediated posttranscriptional mechanisms in human GC. Bmi-1-induced miR-27a and miR-155 were candidate microRNAs identified by microarray analysis and were verified to regulate RKIP. Furthermore, the Bmi-1/miR-27a/RKIP and Bmi-1/miR-155/RKIP signaling axes might be potent targets for novel therapeutic approaches against human GC due to their demonstrated roles in tumor metastasis and drug resistance. Future studies should focus on these aspects.

## Supplementary information

**Additional file 1: Fig. S1** The association between clinical data and Bmi-1 and RKIP. A. qRT-PCR analysis of Bmi-1 and RKIP RNA expression in 15 paired GC tissues (T) and adjacent normal tissue samples (N). B. Western blotting analysis of Bmi-1 and RKIP in 15 paired GC tissues. The definitions of T and N were the same as mentioned in A. C. Kaplan-Meier analysis of the 3-year overall survival of patients with intestinal-type or diffuse-type GC from TCGA. D. Bmi-1, miR-27a and miR-155 were upregulated, while RKIP was downregulated significantly in GC tissues from the TCGA database. *P < 0.05, **P < 0.01. **Fig. S2** Bmi-1 does not upregulate RKIP at the mRNA level nor induce RKIP protein degradation. A. Bmi-1 and RKIP mRNA expression in GES-1 cells overexpressing Bmi-1. *P < 0.05 vs. GES-1-Vector. B. GES-1-Bmi-1 cells and GES-1-Vector cells were subjected to the protein synthesis inhibitor cycloheximide for the indicated period of time. The half-life of RKIP protein in Bmi-1-transduced cells was comparable to that in the control cells, which indicated that Bmi-1 did not induce RKIP protein degradation. **Fig. S3** Quantification of Western blotting assays as well as invasion and migration assays. A. The densitometry analysis of bands from the Western blotting assays in Fig. 2f. ^*^P < 0.05 vs. NC mimic/NC inhibitor. B. The densitometry analysis of bands from the Western blotting assays in Fig. 3a. ^*^P < 0.05 vs. Vector-Ctrl/siNC. C. Analysis of the quantities of invading cells in migration and invasion assays. *P < 0.05 vs. shcon, **P < 0.01 vs. shcon/Vector-Ctrl, ^##^*P* < 0.01 vs. NC mimic/NC inhibitor. **Fig. S4** miR-27a inhibitor and miR-155 inhibitor weakened the effects of Bmi-1 overexpression in functional experiments. A. Bmi-1 upregulation induced gastric cancer cell migration and invasion, which were decreased by the miR-155 inhibitor or miR-27a inhibitor (100 × magnification). B. The reduced ability of cell proliferation due to the transient transfection of the miR-155 inhibitor or miR-27a inhibitor was improved by Bmi-1 overexpression. C. Colony formation assays either in soft agar or on plates showed that the Bmi-1 overexpression group generated more colonies than any other group, and the effect could be reversed by miR-155 inhibitor or miR-27a inhibitor. D. The IC_50_ values of cells treated with 5-Fu or oxaliplatin were detected by CCK8 reagent. The increase in Bmi-1 reduced chemosensitivity, while the miR-155 inhibitor and miR-27a inhibitor lowered the IC_50_. ^*^P < 0.05 vs. Vector-Ctrl, ^#^*P* < 0.05 vs. NC inhibitor. **Fig. S5** Immunohistochemistry of tumors for the detection of Bmi-1, RKIP, Vimentin, Bax and Bcl-2. A. Image from immunohistochemistry of isolated tumors from animals. The animals were subcutaneously implanted with cells stably overexpressing miR-155 or miR-27a and then subjected to intraperitoneal injection of 5-Fu. B. Immunostained sections of different tumors from animals that were implanted with cells stably transfected with shRNA or cotransfected with shRNA and miRNA. Magnification: 200 × . **Supplementary Table S1.** Sequences of primers. **Supplementary Table S2.** All differentially expressed miRNAs.

## Data Availability

The data supporting our findings can be found either in this article or in the supplementary materials.

## Abbreviations

3’UTR: 3′ untranslated region
GC: Gastric cancer
miR-27a: miR-27a-3p
miR-155: miR-155-5p
qRT-PCR: Quantitative real-time polymerase chain reaction
EMT: Epithelial-mesenchymal transition
H&E: Hematoxylin and eosin
Bmi-1: B cell-specific Moloney murine leukemia virus integration site 1
RKIP: Raf kinase inhibitory protein
miRNA: microRNA
EMSA: Electrophoretic mobility shift assay
Bcl-2: B-cell lymphoma-2
Bax: BCL2 Associated X, Apoptosis Regulator
E-cadherin: Cadherin 1
