# Supplementary material for: Bmi-1-induced miR-27a and miR-155 promote tumor metastasis and chemoresistance by targeting RKIP in gastric cancer
Source: Mol Cancer. 2020 Jun 24;19:109. doi: 10.1186/s12943-020-01229-y (PMC7315508; doi:10.1186/s12943-020-01229-y)
Supplement: Supplementary file 1 — Additional file 1: Fig. S1 The association between clinical data and Bmi-1 and RKIP. A. qRT-PCR analysis of Bmi-1 and RKIP RNA expression in 15 paired GC tissues (T) and adjacent normal tissue samples (N). B. Western blotting analysis of Bmi-1 and RKIP in 15 paired GC tissues. The definitions of T and N were the same as mentioned in A. C. Kaplan-Meier analysis of the 3-year overall survival of patients with intestinal-type or diffuse-type GC from TCGA. D. Bmi-1, miR-27a and miR-155 were upregulated, while RKIP was downregulated significantly in GC tissues from the TCGA database. *P < 0.05, **P < 0.01. Fig. S2 Bmi-1 does not upregulate RKIP at the mRNA level nor induce RKIP protein degradation. A. Bmi-1 and RKIP mRNA expression in GES-1 cells overexpressing Bmi-1. *P < 0.05 vs. GES-1-Vector. B. GES-1-Bmi-1 cells and GES-1-Vector cells were subjected to the protein synthesis inhibitor cycloheximide for the indicated period of time. The half-life of RKIP protein in Bmi-1-transduced cells was comparable to that in the control cells, which indicated that Bmi-1 did not induce RKIP protein degradation. Fig. S3 Quantification of Western blotting assays as well as invasion and migration assays. A. The densitometry analysis of bands from the Western blotting assays in Fig. 2f. *P < 0.05 vs. NC mimic/NC inhibitor. B. The densitometry analysis of bands from the Western blotting assays in Fig. 3a. *P < 0.05 vs. Vector-Ctrl/siNC. C. Analysis of the quantities of invading cells in migration and invasion assays. *P < 0.05 vs. shcon, **P < 0.01 vs. shcon/Vector-Ctrl, ##P < 0.01 vs. NC mimic/NC inhibitor. Fig. S4 miR-27a inhibitor and miR-155 inhibitor weakened the effects of Bmi-1 overexpression in functional experiments. A. Bmi-1 upregulation induced gastric cancer cell migration and invasion, which were decreased by the miR-155 inhibitor or miR-27a inhibitor (100 × magnification). B. The reduced ability of cell proliferation due to the transient transfection of the miR-155 inhibit [file 12943_2020_1229_MOESM1_ESM.zip › Marked Supplementary materials.docx]

**Bmi-1-induced miR-27a and miR-155 promote tumor metastasis and chemoresistance by targeting RKIP in gastric cancer.**

Yaqing Li^1,2†^, Zhenfeng Tian^1,2†^, Ying Tan^1,2†^, Guoda Lian^1,2^, Shangxiang Chen^1,2^, Shaojie Chen^1,2^, Jiajia Li^1,3^, Xuanna Li^1,2^, Kaihong Huang^1,2*^, Yinting Chen^1,2*^

**Methods**

**RNA extraction and quantitative real-time PCR analysis**

TRIzol (Invitrogen, Carlsbad, CA, USA) was used to isolate total RNA from cells. For mRNA detection, cDNA for reverse-transcription polymerase chain reaction (RT-PCR) was synthesized with PrimeScript^TM^ RT Master Mix (TaKaRa, Shiga, Japan). For the detection of mature miRNAs, 10 ng of small RNA extracted with TRIzol was reverse transcribed to cDNA using a TaqMan® MicroRNA Reverse Transcription Kit (Applied Biosystems^TM^, Thermo Fisher Scientific, MA, USA). cDNA was used for the amplification of mature miR-27a, miR-155 and an endogenous control (U6) by PCR.

Quantitative real-time PCR assays were performed on a Roche LightCycler 96 Instrument with SYBR Premix Ex Taq II (TaKaRa). The primers can be found in Supplementary Table S1. The qRT-PCR of miRNAs proceeded as follows: 95 °C for 300 s followed by 40 cycles of extension, each consisting of 95 °C for 10 s, 57 °C for 10 s and 72 °C for 15 s. Target genes at the relative transcript level were normalized to the expression of GAPDH or U6 and calculated by the 2^−ΔΔCt^ method. Each experiment was conducted in triplicate, and all experiments were repeated independently 3 times.

**Western blotting analysis**

The cells were collected and lysed in RIPA buffer containing a protease inhibitor cocktail (Roche, Basle, Switzerland). Protein was quantified and separated by 10% SDS-PAGE gels. Then, it was transferred to polyvinylidene fluoride (PVDF) membranes (0.45 µm, Millipore, MA, USA) and immunoblotted with the following primary antibodies: anti-Bmi-1 (1:1000; Cat No. 6964, Cell Signaling Technology, CST, MA, USA), anti-RKIP (1:2000; Cat No. ab2634, Abcam, MA, USA), anti-E-cadherin (1:2000; Cat No. ab40772, Abcam), anti-Vimentin (1:1000; Cat No. 5741, CST), anti-Bax (1:2000; Cat No. ab32503, Abcam), anti-Bcl-2 (1:2000; Cat No. ab32124, Abcam), and anti-GAPDH (1:1000; Cat No. 5174, CST) overnight at 4 °C. The membrane was then washed three times with TBST and incubated with the secondary antibody in blocking buffer at 37 °C for 1 hour. Signal intensity was detected by an enhanced chemiluminescence detection kit (Merck, Darmstadt, Germany). GAPDH was used as the housekeeping gene control. The corresponding densitometry of bands was quantified by ImageJ.

**Cell proliferation assay**

Cell viability was assessed using the MTS Assay Kit according to the Promega protocol or the Cell Counting Kit-8 (CCK8, Dojindo Laboratories, Kumamoto, Japan). Briefly, cells were seeded in 96-well plates (500 per well, or 5 × 10^3^ per well for oxaliplatin and 5-Fu treatment) in triplicate. After a certain incubation time, the original medium was removed, and 0.1 ml of 10% MTS/DMEM solution was added. This was then followed by incubation of the cells in the dark for 3 hours at 37 °C before the absorbance was detected at 492 nm and the IC_50_ was calculated. Each independent experiment was performed three times.

**Colony formation assays**

The colony formation assays were performed according to the protocol described previously. For the plate colony assay, cells were seeded in a 6-well plate at a density of 500 cells per well. In the soft agar colony assay, the 6-well plate was coated with 0.6% bottom agarose in advance, and then 1,000 cells per well were seeded in a 0.3% top agarose layer. After incubation for 12 days, colonies formed on the plates were fixed (with 4% formaldehyde) and stained (with 2% crystal violet). In addition, the colonies growing within the agarose after 14 days were observed and counted using an inverted fluorescence microscope (ECLIPSE Ti, Nikon Corporation, Tokyo, Japan).

**Invasion and migration assays**

BGC823 cells (4 × 10^4^) or SGC7901 cells (3 × 10^4^) were plated per well in a Matrigel-coated 8-µm pore chamber (Corning, New York, USA) with serum-free medium. Medium containing 10% FBS was added to the bottom chamber. The chambers were then incubated in a humid atmosphere of 5% CO_2_ and 95% air at 37 °C for 48 hours. At the end of incubation, cells inside the upper chamber were wiped off with cotton swabs. Subsequently, migrated and invaded cells on the lower membrane surface were fixed with 4% paraformaldehyde/PBS and stained with crystal violet. The number of cells on the underside of each chamber was quantified by counting seven randomly chosen fields (magnification: 100 ×), after which the averages per field were finally calculated. The procedure of the migration assay was similar to that of the invasion assay, except that there was no Matrigel coat and the incubation period was 24 hours.

**Supplementary figure legends**

**Fig. S1** The association between clinical data and Bmi-1 and RKIP. A. qRT-PCR analysis of Bmi-1 and RKIP RNA expression in 15 paired GC tissues (T) and adjacent normal tissue samples (N). B. Western blotting analysis of Bmi-1 and RKIP in 15 paired GC tissues. The definitions of T and N were the same as mentioned in A. C. Kaplan-Meier analysis of the 3-year overall survival of patients with intestinal-type or diffuse-type GC from TCGA. D. Bmi-1, miR-27a and miR-155 were upregulated, while RKIP was downregulated significantly in GC tissues from the TCGA database. **P* < 0.05, ***P* < 0.01.

**Fig. S2** Bmi-1 does not upregulate RKIP at the mRNA level nor induce RKIP protein degradation. A. Bmi-1 and RKIP mRNA expression in GES-1 cells overexpressing Bmi-1. **P* < 0.05 *vs*. GES-1-Vector. B. GES-1-Bmi-1 cells and GES-1-Vector cells were subjected to the protein synthesis inhibitor cycloheximide for the indicated period of time. The half-life of RKIP protein in Bmi-1-transduced cells was comparable to that in the control cells, which indicated that Bmi-1 did not induce RKIP protein degradation.

**Fig. S3** Quantification of Western blotting assays as well as invasion and migration assays. A. The densitometry analysis of bands from the Western blotting assays in Fig. 2F. ^*^*P* < 0.05 *vs.* NC mimic/NC inhibitor. B. The densitometry analysis of bands from the Western blotting assays in Fig. 3A. ^*^*P* < 0.05 *vs.* Vector-Ctrl/siNC. C. Analysis of the quantities of invading cells in migration and invasion assays. **P* < 0.05 *vs.* shcon, ***P* < 0.01 *vs.* shcon/Vector-Ctrl, ^##^*P* < 0.01 *vs*. NC mimic/NC inhibitor.

**Fig. S4** miR-27a inhibitor and miR-155 inhibitor weakened the effects of Bmi-1 overexpression in functional experiments. A. Bmi-1 upregulation induced gastric cancer cell migration and invasion, which were decreased by the miR-155 inhibitor or miR-27a inhibitor (100 × magnification). B. The reduced ability of cell proliferation due to the transient transfection of the miR-155 inhibitor or miR-27a inhibitor was improved by Bmi-1 overexpression. C. Colony formation assays either in soft agar or on plates showed that the Bmi-1 overexpression group generated more colonies than any other group, and the effect could be reversed by miR-155 inhibitor or miR-27a inhibitor. D. The IC_50_ values of cells treated with 5-Fu or oxaliplatin were detected by CCK8 reagent. The increase in Bmi-1 reduced chemosensitivity, while the miR-155 inhibitor and miR-27a inhibitor lowered the IC_50_. ^*^*P* < 0.05 *vs.* Vector-Ctrl, ^#^*P* < 0.05 *vs.* NC inhibitor.

**Fig. S5** Immunohistochemistry of tumors for the detection of Bmi-1, RKIP, Vimentin, Bax and Bcl-2. A. Image from immunohistochemistry of isolated tumors from animals. The animals were subcutaneously implanted with cells stably overexpressing miR-155 or miR-27a and then subjected to intraperitoneal injection of 5-Fu. B. Immunostained sections of different tumors from animals that were implanted with cells stably transfected with shRNA or cotransfected with shRNA and miRNA. Magnification: 200×.

**Supplementary Table S1. Sequences of primers**

| Name | sequence |
| --- | --- |
| **For RT-PCR** |  |
| Bmi-1-forward | 5’-CAGGGTACTTCATTGATGCCACAAC-3’ |
| Bmi-1-reverse | 5’-GCTGGTCTCCAGGTAACGAACAATA-3’ |
| RKIP-forward | 5’-AAGCTCTACACCTTGGTCCTGACA-3’ |
| RKIP-reverse | 5’-TGCTCGTAAACCAGCCAGACA-3’ |
| GAPDH-forward | 5’-GCACCGTCAAGGCTGAGAAC-3’ |
| GAPDH-reverse | 5’-TGGTGAAGACGCCAGTGGA-3’ |
| E-cadherin-forward | 5’-ATTCTGATTCTGCTGCTCTTG-3’ |
| E-cadherin-reverse | 5’-AGTCCTGGTCCTCTTCTCC-3’ |
| vimentin-forward | 5’-CCAAACTTTTCCTCCCTGAACC-3’ |
| vimentin-reverse | 5’-GTGATGCTGAGAAGTTTCGTTGA-3’ |
| hsa-miR-27a  -stem-loop | 5’-GTCGTATCCAGTGCAGGGTCCGAGGTATTCGCACTGGATACGACGCGGAA-3’, |
| hsa-miR-155  -stem-loop | 5’-GTCGTATCCAGTGCAGGGTCCGAGGTATTCGCACTGGATACGACACCCCT-3’ |
| U6-stem-loop | 5’-GTCGTATCCAGTGCAGGGTCCGAGGTATTCGCACTGGATACGACAAAATATGGAAC-3’ |
| hsa-miR-27a-forward | 5’-GGCGGTTCACAGTGGCTAAG-3’, |
| hsa-miR-155-forward | 5’- GCGCGGCTTAATGCTAATCGTG-3’ |
| U6-forward | 5’-TGCGGG TGCTCGCTTCGGCAGC-3’ |
| miRNA universal-reverse | 5’-CCAGTGCAGGGTCCGAGGT-3’ |
| **For shRNA** |  |
| shBmi-1 sense | 5’-UGUCUACAUUCCUUCUGUATT-3’ |
| shBmi-1 antisense | 5’-UACAGAAGGAAUGUAGACATT-3’ |
| **For EMSA** |  |
| hsa-miR-27a target/RKIP-Target-1 | 5’-GGGGUAUUUUGGUACUGUGAU-3’ |
| hsa-miR-155 target/RKIP-Target-2 | 5’-AGUUGCUGAAUGUUGCAUUAAU-3’ |

Abbreviations: Bmi-1, B cell-specific Moloney murine leukemia virus integration site 1; RKIP, Raf kinase inhibitory protein; E-cadherin, Cadherin 1.

**Supplementary Table S2.** **All differentially expressed miRNAs**

| **microRNA Name** | **Fold change** | **Foreground** | | **Foreground-Background** | | **Normalized** | |
| --- | --- | --- | --- | --- | --- | --- | --- |
|  | **B1 vs V1** | **V1** | **B1** | **V1** | **B1** | **V1** | **B1** |
| hsa-miR-31-5p | 2.110585 | 158 | 265.5 | 67 | 173.5 | 0.180471 | 0.3809 |
| hsa-let-7b-5p | 4.754391 | 107.5 | 179 | 15 | 87.5 | 0.040404 | 0.192097 |
| hsa-miR-920 | 2.264271 | 259 | 555 | 164.5 | 457 | 0.443098 | 1.003293 |
| hsa-miR-5580-5p | 2.991275 | 245.5 | 639 | 145.5 | 534 | 0.391919 | 1.172338 |
| hsa-miR-4503 | 5.214493 | 144.5 | 386.5 | 46.5 | 297.5 | 0.125253 | 0.653128 |
| hsa-miR-27a-3p | 2.05141 | 206 | 386.5 | 118 | 297 | 0.317845 | 0.652031 |
| hsa-let-7g-5p | 4.576754 | 100.5 | 158.5 | 13 | 73 | 0.035017 | 0.160263 |
| hsa-miR-4764-5p | 18.78293 | 105 | 342.5 | 11 | 253.5 | 0.02963 | 0.556531 |
| hsa-miR-761 | 3.677735 | 178.5 | 459 | 81 | 365.5 | 0.218182 | 0.802415 |
| hsa-miR-155-5p | 5.798416 | 112.5 | 212 | 17.5 | 124.5 | 0.047138 | 0.273326 |
| hsa-miR-33a-5p | 3.336564 | 119 | 158.5 | 16 | 65.5 | 0.043098 | 0.143798 |
| hsa-miR-4725-3p | 15.65914 | 122 | 553.5 | 23.5 | 451.5 | 0.0633 | 0.991218 |
| hsa-miR-1285-3p | 4.204857 | 112 | 198.5 | 22 | 113.5 | 0.059259 | 0.249177 |
| hsa-miR-1273e | 21.48738 | 106 | 231.5 | 5.5 | 145 | 0.014815 | 0.318332 |
| hsa-miR-934 | 9.273816 | 109.5 | 297.5 | 18.5 | 210.5 | 0.049832 | 0.46213 |
| hsa-miR-1184 | 4.193504 | 185 | 565 | 93 | 478.5 | 0.250505 | 1.050494 |
| hsa-miR-498 | 2.651075 | 331.5 | 843 | 231.5 | 753 | 0.623569 | 1.653128 |
| hsa-miR-4784 | 6.854339 | 134 | 348.5 | 30.5 | 256.5 | 0.082155 | 0.563117 |
| hsa-miR-3591-5p | 2.238025 | 447 | 1016.5 | 336.5 | 924 | 0.906397 | 2.02854 |
| hsa-miR-890 | 41.91626 | 109 | 812 | 14 | 720 | 0.03771 | 1.580681 |
| hsa-miR-363-5p | 2.442623 | 250 | 572 | 163.5 | 490 | 0.440404 | 1.075741 |
| hsa-let-7i-5p | 58.27525 | 85.5 | 161.5 | 1 | 71.5 | 0.002694 | 0.15697 |
| hsa-miR-22-3p | 3.458092 | 120 | 233 | 35 | 148.5 | 0.094276 | 0.326015 |
| hsa-let-7c | 3.803513 | 98.5 | 152.5 | 12 | 56 | 0.032323 | 0.122942 |
| hsa-miR-3924 | 8.103958 | 166.5 | 894.5 | 79 | 785.5 | 0.212795 | 1.724479 |
| kshv-miR-K12-5 | 3.389367 | 128 | 258.5 | 41 | 170.5 | 0.110438 | 0.374314 |
| hsa-miR-29a-3p | 2.899584 | 193.5 | 473 | 108.5 | 386 | 0.292256 | 0.84742 |
| hsa-miR-5689 | 2.561549 | 130.5 | 228 | 45.5 | 143 | 0.122559 | 0.313941 |
| hsa-miR-4429 | 15.04686 | 103 | 324.5 | 13 | 240 | 0.035017 | 0.526894 |
| hsa-miR-4788 | 2.270061 | 311 | 675.5 | 216.5 | 603 | 0.583165 | 1.32382 |
| hsa-miR-4658 | 3.60715 | 137.5 | 315 | 50.5 | 223.5 | 0.136027 | 0.49067 |
| hsa-miR-3935 | 4.802566 | 260.5 | 1112.5 | 172 | 1013.5 | 0.4633 | 2.225027 |
| hsa-miR-513c-3p | 7.464036 | 100.5 | 180.5 | 9.5 | 87 | 0.025589 | 0.190999 |
| hsa-miR-4709-3p | 2.105027 | 712.5 | 1711 | 625.5 | 1615.5 | 1.684848 | 3.546652 |
| hsa-miR-4284 | 2.239518 | 1807.5 | 4815 | 1718.5 | 4722 | 4.628956 | 10.36663 |
| hsa-miR-4677-3p | 2.574069 | 413 | 1121 | 325.5 | 1028 | 0.876768 | 2.256861 |
| hsa-miR-518e-5pmiR-519a-5p/hsa-miR-519b-5p/hsa-miR-519c-5p/hsa-miR-522-5p/hsa-miR-523-5p | 14.48261 | 95.5 | 433 | 19.5 | 346.5 | 0.052525 | 0.760703 |
| hsa-miR-22-5p | 11.17659 | 139 | 833 | 54 | 740.5 | 0.145455 | 1.625686 |
| hsa-miR-3607-5p | 2.029605 | 114.5 | 152.5 | 25.5 | 63.5 | 0.068687 | 0.139407 |
| hsa-miR-552 | 3.183744 | 118 | 227.5 | 32 | 125 | 0.086195 | 0.274424 |
| hsa-miR-93-5p | 17.31957 | 89.5 | 190.5 | 4 | 85 | 0.010774 | 0.186608 |
| kshv-miR-K12-1 | 6.048299 | 232 | 1192 | 148.5 | 1102 | 0.4 | 2.419319 |
| hsa-miR-516b-5p | 9.821213 | 149 | 819 | 60 | 723 | 0.161616 | 1.587267 |
| hsa-miR-584-5p | 3.15175 | 186.5 | 503.5 | 101.5 | 392.5 | 0.273401 | 0.86169 |
| hsa-miR-4471 | 70.0933 | 87 | 312 | 2.5 | 215 | 0.006734 | 0.472009 |
| hsa-miR-585 | 6.874486 | 186 | 870 | 95.5 | 805.5 | 0.257239 | 1.768386 |
| hsa-miR-1321 | 2.128398 | 362.5 | 801.5 | 280.5 | 732.5 | 0.755556 | 1.608123 |
| hsa-miR-451b | 2.402499 | 157 | 313.5 | 76.5 | 225.5 | 0.206061 | 0.49506 |
| hsa-miR-9-3p | 3.77579 | 160.5 | 428.5 | 73.5 | 340.5 | 0.19798 | 0.74753 |
| hsa-miR-4329 | 4.965971 | 314.5 | 1606 | 247.5 | 1508 | 0.666667 | 3.310648 |
| hsa-miR-4765 | 2.292855 | 179 | 350.5 | 91 | 256 | 0.245118 | 0.56202 |
| hsa-miR-181a-2-3p | 0.071736 | 359 | 112.5 | 267 | 23.5 | 0.719192 | 0.051592 |
| hsv1-miR-H6-3p | 0.467838 | 230 | 162 | 138.5 | 79.5 | 0.373064 | 0.174533 |
| hsa-miR-501-5p | 0.490532 | 142.5 | 121 | 54 | 32.5 | 0.145455 | 0.07135 |
| hsv2-miR-H6* | 0.422696 | 454.5 | 280.5 | 362.5 | 188 | 0.976431 | 0.412733 |
| hsa-miR-4475 | 0.427182 | 1676.5 | 920.5 | 1585.5 | 831 | 4.270707 | 1.824369 |
| hsa-miR-4726-5p | 0.476126 | 2962.5 | 1769.5 | 2869 | 1676 | 7.727946 | 3.679473 |
| hsa-miR-1204 | 0.033495 | 170.5 | 98 | 73 | 3 | 0.196633 | 0.006586 |
| hsa-miR-302a-3p | 0.435632 | 2681 | 1478.5 | 2587.5 | 1383 | 6.969697 | 3.036224 |
| hsa-miR-4279 | 0.498598 | 446.5 | 303.5 | 349 | 213.5 | 0.940067 | 0.468716 |
| hsv2-miR-H7-5p | 0.279089 | 755.5 | 320.5 | 660 | 226 | 1.777778 | 0.496158 |
| hsa-miR-483-3p | 0.477033 | 740 | 463 | 639 | 374 | 1.721212 | 0.821076 |
| hsa-miR-4653-3p | 0.051748 | 162 | 94 | 63 | 4 | 0.169697 | 0.008782 |
| hsa-miR-2682-3p | 0.054078 | 308.5 | 108.5 | 211 | 14 | 0.56835 | 0.030735 |
| hsa-let-7a-2-3p | 0.177461 | 537.5 | 184 | 445.5 | 97 | 1.2 | 0.212953 |
| hsa-miR-1908 | 0.36961 | 306 | 188.5 | 215 | 97.5 | 0.579125 | 0.21405 |
| hsa-miR-3667-5p | 0.459745 | 2166.5 | 1256 | 2079.5 | 1173 | 5.601347 | 2.575192 |
| hsa-miR-4450 | 0.336999 | 2649.5 | 1148 | 2560 | 1058.5 | 6.895623 | 2.32382 |
| hsa-miR-4468 | 0.40782 | 2119.5 | 1102 | 2031.5 | 1016.5 | 5.472054 | 2.231614 |
| ebv-miR-BART19-3p | 0.346756 | 594.5 | 308 | 503 | 214 | 1.354882 | 0.469813 |
| hsa-miR-664-3p | 0.2071 | 161 | 111 | 61 | 15.5 | 0.16431 | 0.034029 |
| hsa-miR-519e-5p | 0.409547 | 191 | 138 | 100.5 | 50.5 | 0.270707 | 0.110867 |
| hsa-miR-3157-3p | 0.157528 | 153.5 | 106 | 59.5 | 11.5 | 0.160269 | 0.025247 |
| hsa-miR-4775 | 0.20376 | 160.5 | 109.5 | 64 | 16 | 0.172391 | 0.035126 |
| hsa-miR-4456 | 0.49156 | 18665.5 | 11295 | 18567 | 11198 | 50.01212 | 24.58397 |
| hsa-miR-4732-3p | 0.23564 | 237.5 | 135 | 147 | 42.5 | 0.39596 | 0.093304 |
| hsa-miR-4508 | 0.307035 | 240 | 150 | 146 | 55 | 0.393266 | 0.120746 |
| hsa-miR-125a-5p | 0.494103 | 273.5 | 198.5 | 176.5 | 107 | 0.475421 | 0.234907 |
| hsa-miR-3152-5p | 0.353509 | 2601 | 1180.5 | 2505 | 1086.5 | 6.747475 | 2.385291 |
| ebv-miR-BART13 | 0.342316 | 215.5 | 138.5 | 125 | 52.5 | 0.3367 | 0.115258 |
| hsa-miR-935 | 0.128525 | 479.5 | 148.5 | 390 | 61.5 | 1.050505 | 0.135016 |
| hsa-miR-340-3p | 0.157041 | 812 | 225 | 724 | 139.5 | 1.950168 | 0.306257 |
| hsa-miR-149-3p | 0.49058 | 240.5 | 178.5 | 157 | 94.5 | 0.422896 | 0.207464 |
| hsa-miR-181d | 0.25667 | 266.5 | 141 | 181 | 57 | 0.487542 | 0.125137 |
| hsa-miR-130b-3p | 0.094425 | 176.5 | 101.5 | 82 | 9.5 | 0.220875 | 0.020856 |
| ebv-miR-BHRF1-1 | 0.19529 | 300.5 | 143.5 | 204.5 | 49 | 0.550842 | 0.107574 |
| hsa-miR-423-3p | 0.190019 | 358.5 | 159.5 | 259.5 | 60.5 | 0.69899 | 0.132821 |
| hsa-miR-15a-3p | 0.208854 | 170.5 | 110.5 | 80 | 20.5 | 0.215488 | 0.045005 |
| hsa-miR-4431 | 0.242993 | 175.5 | 114.5 | 80.5 | 24 | 0.216835 | 0.052689 |
| hsa-miR-4669 | 0.375916 | 225 | 148.5 | 122.5 | 56.5 | 0.329966 | 0.12404 |
| hsa-miR-4804-3p | 0.11979 | 446 | 140 | 347 | 51 | 0.93468 | 0.111965 |
| hsa-miR-4436b-5p | 0.048902 | 150 | 94.5 | 50 | 3 | 0.13468 | 0.006586 |
| hsa-miR-4750 | 0.282485 | 275 | 150 | 176 | 61 | 0.474074 | 0.133919 |
| hsa-miR-564 | 0.240584 | 177 | 117 | 83 | 24.5 | 0.223569 | 0.053787 |
| hsa-miR-615-3p | 0.275589 | 445.5 | 209 | 347.5 | 117.5 | 0.936027 | 0.257958 |
| hsa-miR-5196-3p | 0.458143 | 257.5 | 176 | 161 | 90.5 | 0.43367 | 0.198683 |
| hsv1-miR-H8* | 0.054336 | 163 | 94 | 75 | 5 | 0.20202 | 0.010977 |
| hsa-miR-2355-3p | 0.323573 | 1421.5 | 618.5 | 1335 | 530 | 3.59596 | 1.163557 |
| hsa-miR-138-2-3p | 0.451782 | 222 | 163 | 133.5 | 74 | 0.359596 | 0.162459 |
| hsa-miR-630 | 0.463256 | 977.5 | 592.5 | 892 | 507 | 2.402694 | 1.113063 |
| kshv-miR-K12-6-3p | 0.144699 | 638.5 | 186.5 | 552 | 98 | 1.486869 | 0.215148 |
| hsa-miR-644b-3p | 0.462785 | 1211 | 729.5 | 1124.5 | 638.5 | 3.028956 | 1.401756 |
| hsa-miR-4756-3p | 0.151338 | 311 | 129 | 223.5 | 41.5 | 0.60202 | 0.091109 |
| hsa-miR-4639-3p | 0.441777 | 3618 | 2000 | 3533 | 1915 | 9.516498 | 4.204171 |
| hsa-miR-4467 | 0.418332 | 1781 | 957 | 1696 | 870.5 | 4.56835 | 1.911087 |
| hsa-miR-4799-3p | 0.079051 | 234.5 | 100.5 | 149.5 | 14.5 | 0.402694 | 0.031833 |
| hsa-miR-374c-3p | 0.417682 | 493.5 | 295.5 | 401 | 205.5 | 1.080135 | 0.451153 |
| hsa-miR-660-3p | 0.320069 | 1386.5 | 595 | 1288.5 | 506 | 3.470707 | 1.110867 |
| hsa-miR-374b-3p | 0.330813 | 3092.5 | 1311 | 3007 | 1220.5 | 8.099663 | 2.679473 |
| hsa-let-7e-5p | 0.459684 | 3043.5 | 1767.5 | 2953 | 1665.5 | 7.954209 | 3.656422 |
| hsa-miR-4258 | 0.304753 | 145 | 108 | 57.5 | 21.5 | 0.154882 | 0.047201 |
| hsa-miR-4780 | 0.424535 | 2935.5 | 1585 | 2850 | 1484.5 | 7.676768 | 3.259056 |
| hsa-miR-4708-3p | 0.461119 | 1366 | 814 | 1273.5 | 720.5 | 3.430303 | 1.581778 |
| hsa-miR-4473 | 0.393687 | 468.5 | 268.5 | 383 | 185 | 1.03165 | 0.406147 |
| hsa-miR-485-3p | 0.339764 | 907.5 | 433 | 824 | 343.5 | 2.219529 | 0.754116 |
| hsa-miR-4682 | 0.341644 | 533 | 285.5 | 448.5 | 188 | 1.208081 | 0.412733 |
| hsa-miR-5571-5p | 0.371025 | 150.5 | 122.5 | 67 | 30.5 | 0.180471 | 0.066959 |
| hsa-miR-5699 | 0.253122 | 3231.5 | 1063.5 | 3147.5 | 977.5 | 8.478114 | 2.145993 |
| hsa-miR-371b-5p | 0.285754 | 2154 | 824 | 2075 | 727.5 | 5.589226 | 1.597146 |
| hsa-miR-323a-3p | 0.091361 | 262.5 | 108 | 169.5 | 19 | 0.456566 | 0.041712 |
| hsa-miR-550b-3p | 0.245828 | 359.5 | 181 | 278.5 | 84 | 0.750168 | 0.184413 |
| hsa-miR-378a-3p | 0.428968 | 482 | 296.5 | 389.5 | 205 | 1.049158 | 0.450055 |
| hsa-miR-214-3p | 0.269871 | 385 | 184 | 300.5 | 99.5 | 0.809428 | 0.218441 |

# Condition pairs: GES-1-Bmi-1 #1 *vs* GES-1-Vector (B1 *vs* V1)

# Fold change cut-off: 2.0.

# Column "Fold change" contains the ratio of normalized intensities between both conditions.

# Column "Foreground" indicates the foreground intensity of the miRNA.

# Column "Foreground-Background" indicates the signal of the miRNA after background correction.

# Column "Normalized" indicates the normalized signal of the miRNA. We used the median normalization method for array normalization.
