# Supplementary figures and images for: Bmi-1-induced miR-27a and miR-155 promote tumor metastasis and chemoresistance by targeting RKIP in gastric cancer
Source: Mol Cancer. 2020 Jun 24;19:109. doi: 10.1186/s12943-020-01229-y (PMC7315508; doi:10.1186/s12943-020-01229-y)

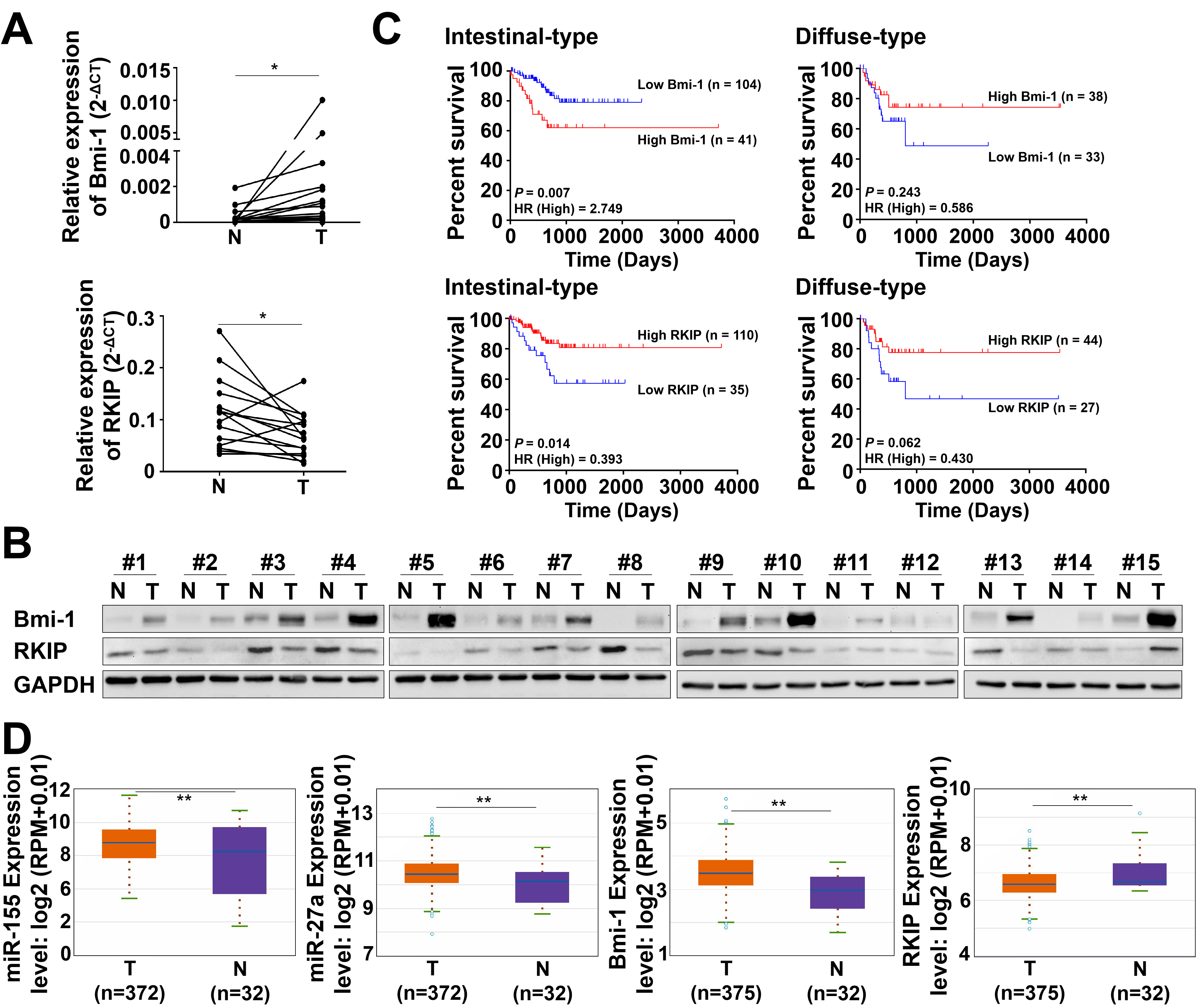

Supplement: Supplementary file 1 — Additional file 1: Fig. S1 The association between clinical data and Bmi-1 and RKIP. A. qRT-PCR analysis of Bmi-1 and RKIP RNA expression in 15 paired GC tissues (T) and adjacent normal tissue samples (N). B. Western blotting analysis of Bmi-1 and RKIP in 15 paired GC tissues. The definitions of T and N were the same as mentioned in A. C. Kaplan-Meier analysis of the 3-year overall survival of patients with intestinal-type or diffuse-type GC from TCGA. D. Bmi-1, miR-27a and miR-155 were upregulated, while RKIP was downregulated significantly in GC tissues from the TCGA database. *P < 0.05, **P < 0.01. Fig. S2 Bmi-1 does not upregulate RKIP at the mRNA level nor induce RKIP protein degradation. A. Bmi-1 and RKIP mRNA expression in GES-1 cells overexpressing Bmi-1. *P < 0.05 vs. GES-1-Vector. B. GES-1-Bmi-1 cells and GES-1-Vector cells were subjected to the protein synthesis inhibitor cycloheximide for the indicated period of time. The half-life of RKIP protein in Bmi-1-transduced cells was comparable to that in the control cells, which indicated that Bmi-1 did not induce RKIP protein degradation. Fig. S3 Quantification of Western blotting assays as well as invasion and migration assays. A. The densitometry analysis of bands from the Western blotting assays in Fig. 2f. *P < 0.05 vs. NC mimic/NC inhibitor. B. The densitometry analysis of bands from the Western blotting assays in Fig. 3a. *P < 0.05 vs. Vector-Ctrl/siNC. C. Analysis of the quantities of invading cells in migration and invasion assays. *P < 0.05 vs. shcon, **P < 0.01 vs. shcon/Vector-Ctrl, ##P < 0.01 vs. NC mimic/NC inhibitor. Fig. S4 miR-27a inhibitor and miR-155 inhibitor weakened the effects of Bmi-1 overexpression in functional experiments. A. Bmi-1 upregulation induced gastric cancer cell migration and invasion, which were decreased by the miR-155 inhibitor or miR-27a inhibitor (100 × magnification). B. The reduced ability of cell proliferation due to the transient transfection of the miR-155 inhibit [file 12943_2020_1229_MOESM1_ESM.zip › Figure S1.tif]

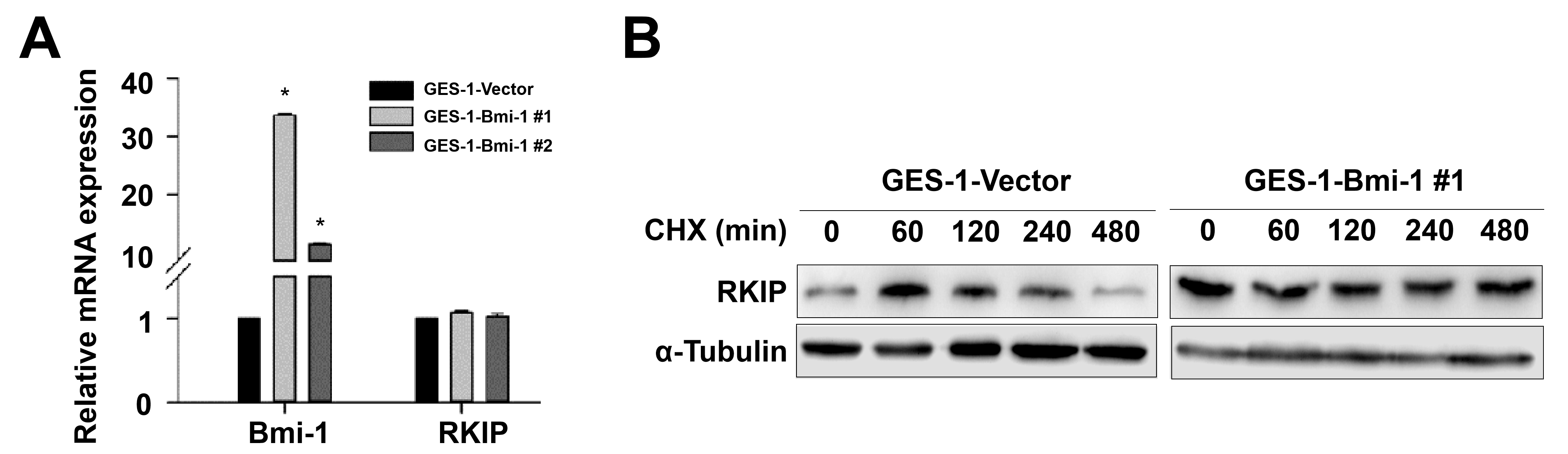

Supplement: Supplementary file 1 — Additional file 1: Fig. S1 The association between clinical data and Bmi-1 and RKIP. A. qRT-PCR analysis of Bmi-1 and RKIP RNA expression in 15 paired GC tissues (T) and adjacent normal tissue samples (N). B. Western blotting analysis of Bmi-1 and RKIP in 15 paired GC tissues. The definitions of T and N were the same as mentioned in A. C. Kaplan-Meier analysis of the 3-year overall survival of patients with intestinal-type or diffuse-type GC from TCGA. D. Bmi-1, miR-27a and miR-155 were upregulated, while RKIP was downregulated significantly in GC tissues from the TCGA database. *P < 0.05, **P < 0.01. Fig. S2 Bmi-1 does not upregulate RKIP at the mRNA level nor induce RKIP protein degradation. A. Bmi-1 and RKIP mRNA expression in GES-1 cells overexpressing Bmi-1. *P < 0.05 vs. GES-1-Vector. B. GES-1-Bmi-1 cells and GES-1-Vector cells were subjected to the protein synthesis inhibitor cycloheximide for the indicated period of time. The half-life of RKIP protein in Bmi-1-transduced cells was comparable to that in the control cells, which indicated that Bmi-1 did not induce RKIP protein degradation. Fig. S3 Quantification of Western blotting assays as well as invasion and migration assays. A. The densitometry analysis of bands from the Western blotting assays in Fig. 2f. *P < 0.05 vs. NC mimic/NC inhibitor. B. The densitometry analysis of bands from the Western blotting assays in Fig. 3a. *P < 0.05 vs. Vector-Ctrl/siNC. C. Analysis of the quantities of invading cells in migration and invasion assays. *P < 0.05 vs. shcon, **P < 0.01 vs. shcon/Vector-Ctrl, ##P < 0.01 vs. NC mimic/NC inhibitor. Fig. S4 miR-27a inhibitor and miR-155 inhibitor weakened the effects of Bmi-1 overexpression in functional experiments. A. Bmi-1 upregulation induced gastric cancer cell migration and invasion, which were decreased by the miR-155 inhibitor or miR-27a inhibitor (100 × magnification). B. The reduced ability of cell proliferation due to the transient transfection of the miR-155 inhibit [file 12943_2020_1229_MOESM1_ESM.zip › Figure S2.tif]

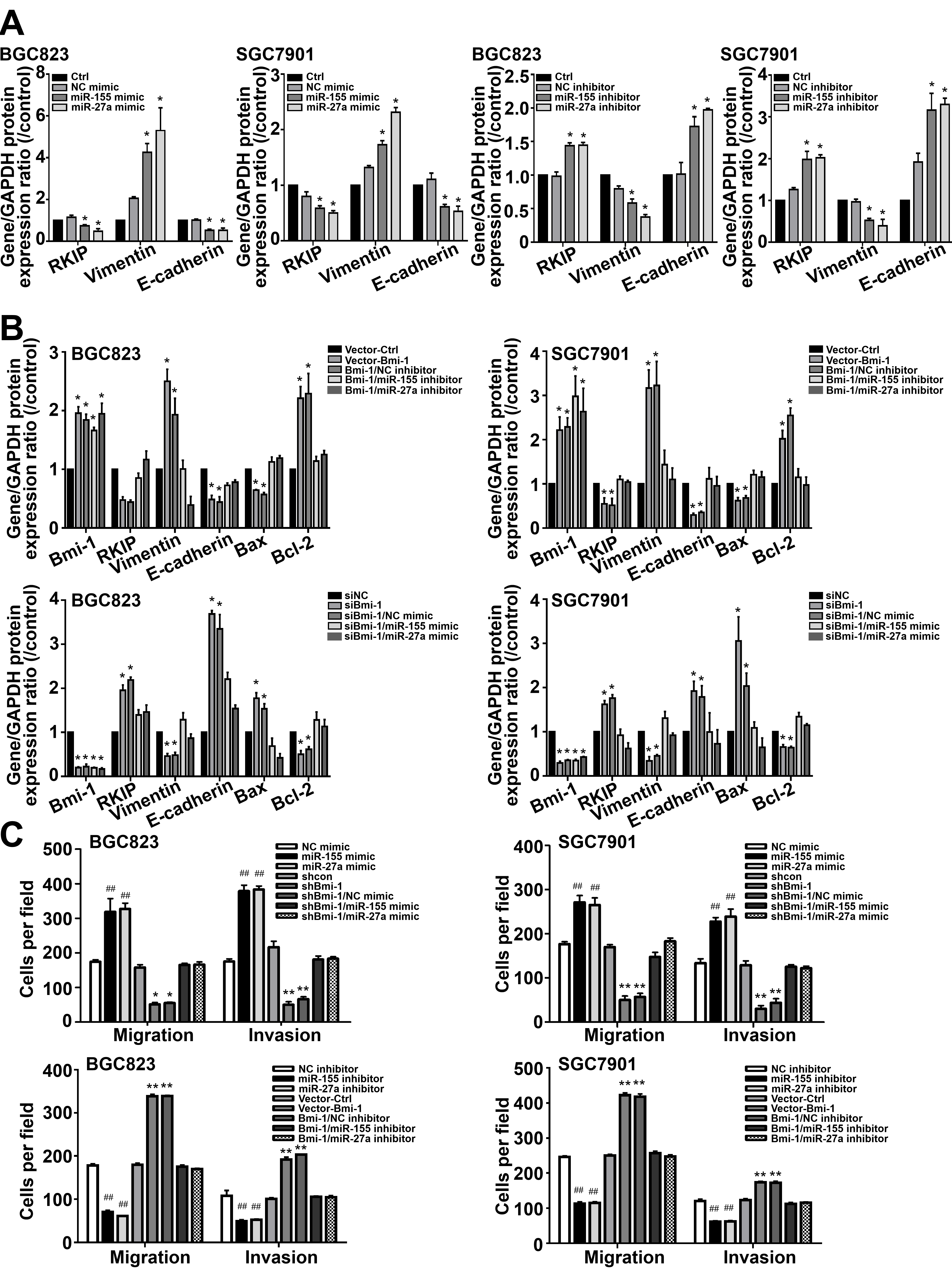

Supplement: Supplementary file 1 — Additional file 1: Fig. S1 The association between clinical data and Bmi-1 and RKIP. A. qRT-PCR analysis of Bmi-1 and RKIP RNA expression in 15 paired GC tissues (T) and adjacent normal tissue samples (N). B. Western blotting analysis of Bmi-1 and RKIP in 15 paired GC tissues. The definitions of T and N were the same as mentioned in A. C. Kaplan-Meier analysis of the 3-year overall survival of patients with intestinal-type or diffuse-type GC from TCGA. D. Bmi-1, miR-27a and miR-155 were upregulated, while RKIP was downregulated significantly in GC tissues from the TCGA database. *P < 0.05, **P < 0.01. Fig. S2 Bmi-1 does not upregulate RKIP at the mRNA level nor induce RKIP protein degradation. A. Bmi-1 and RKIP mRNA expression in GES-1 cells overexpressing Bmi-1. *P < 0.05 vs. GES-1-Vector. B. GES-1-Bmi-1 cells and GES-1-Vector cells were subjected to the protein synthesis inhibitor cycloheximide for the indicated period of time. The half-life of RKIP protein in Bmi-1-transduced cells was comparable to that in the control cells, which indicated that Bmi-1 did not induce RKIP protein degradation. Fig. S3 Quantification of Western blotting assays as well as invasion and migration assays. A. The densitometry analysis of bands from the Western blotting assays in Fig. 2f. *P < 0.05 vs. NC mimic/NC inhibitor. B. The densitometry analysis of bands from the Western blotting assays in Fig. 3a. *P < 0.05 vs. Vector-Ctrl/siNC. C. Analysis of the quantities of invading cells in migration and invasion assays. *P < 0.05 vs. shcon, **P < 0.01 vs. shcon/Vector-Ctrl, ##P < 0.01 vs. NC mimic/NC inhibitor. Fig. S4 miR-27a inhibitor and miR-155 inhibitor weakened the effects of Bmi-1 overexpression in functional experiments. A. Bmi-1 upregulation induced gastric cancer cell migration and invasion, which were decreased by the miR-155 inhibitor or miR-27a inhibitor (100 × magnification). B. The reduced ability of cell proliferation due to the transient transfection of the miR-155 inhibit [file 12943_2020_1229_MOESM1_ESM.zip › Figure S3.tif]

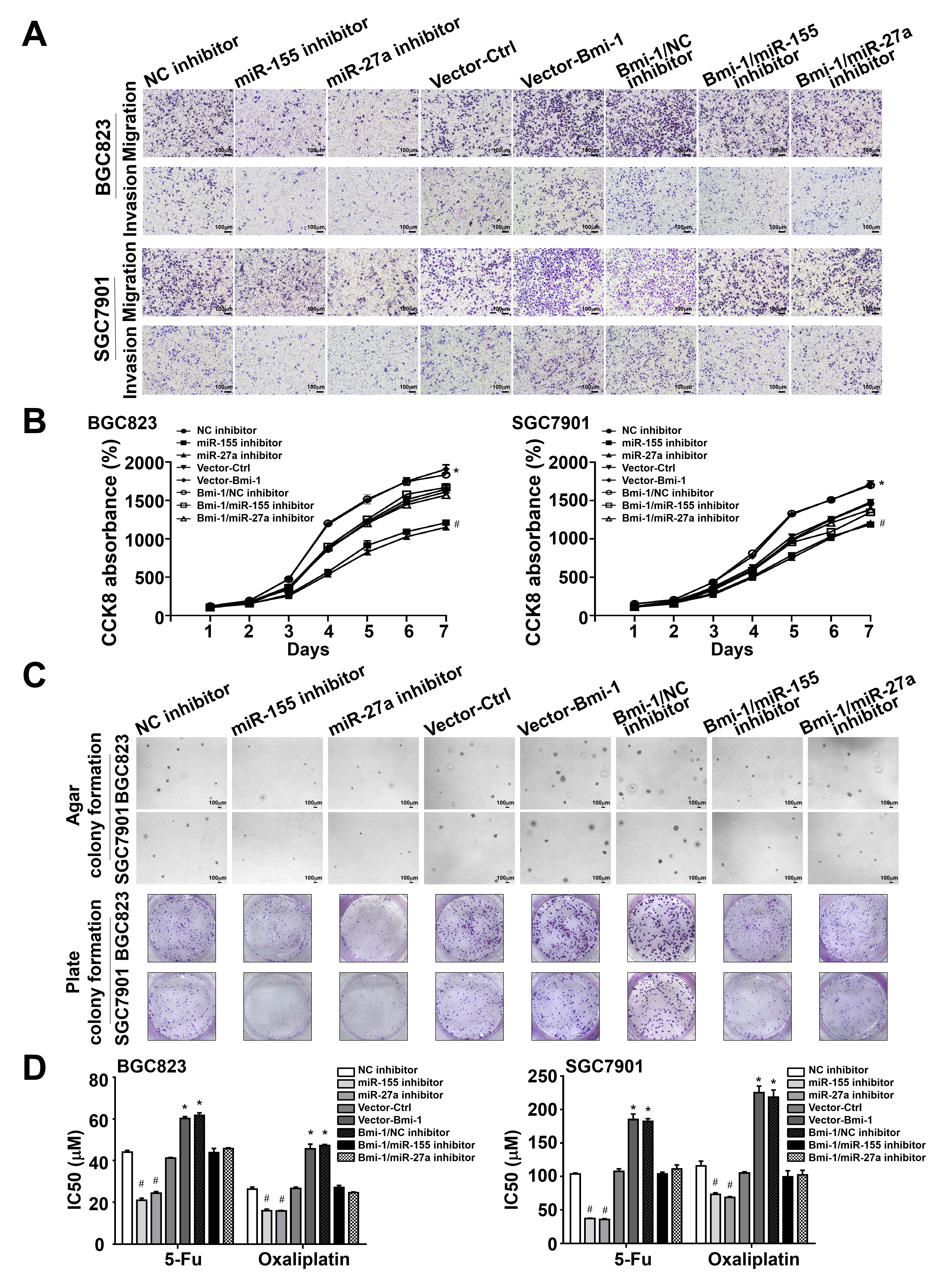

Supplement: Supplementary file 1 — Additional file 1: Fig. S1 The association between clinical data and Bmi-1 and RKIP. A. qRT-PCR analysis of Bmi-1 and RKIP RNA expression in 15 paired GC tissues (T) and adjacent normal tissue samples (N). B. Western blotting analysis of Bmi-1 and RKIP in 15 paired GC tissues. The definitions of T and N were the same as mentioned in A. C. Kaplan-Meier analysis of the 3-year overall survival of patients with intestinal-type or diffuse-type GC from TCGA. D. Bmi-1, miR-27a and miR-155 were upregulated, while RKIP was downregulated significantly in GC tissues from the TCGA database. *P < 0.05, **P < 0.01. Fig. S2 Bmi-1 does not upregulate RKIP at the mRNA level nor induce RKIP protein degradation. A. Bmi-1 and RKIP mRNA expression in GES-1 cells overexpressing Bmi-1. *P < 0.05 vs. GES-1-Vector. B. GES-1-Bmi-1 cells and GES-1-Vector cells were subjected to the protein synthesis inhibitor cycloheximide for the indicated period of time. The half-life of RKIP protein in Bmi-1-transduced cells was comparable to that in the control cells, which indicated that Bmi-1 did not induce RKIP protein degradation. Fig. S3 Quantification of Western blotting assays as well as invasion and migration assays. A. The densitometry analysis of bands from the Western blotting assays in Fig. 2f. *P < 0.05 vs. NC mimic/NC inhibitor. B. The densitometry analysis of bands from the Western blotting assays in Fig. 3a. *P < 0.05 vs. Vector-Ctrl/siNC. C. Analysis of the quantities of invading cells in migration and invasion assays. *P < 0.05 vs. shcon, **P < 0.01 vs. shcon/Vector-Ctrl, ##P < 0.01 vs. NC mimic/NC inhibitor. Fig. S4 miR-27a inhibitor and miR-155 inhibitor weakened the effects of Bmi-1 overexpression in functional experiments. A. Bmi-1 upregulation induced gastric cancer cell migration and invasion, which were decreased by the miR-155 inhibitor or miR-27a inhibitor (100 × magnification). B. The reduced ability of cell proliferation due to the transient transfection of the miR-155 inhibit [file 12943_2020_1229_MOESM1_ESM.zip › Figure S4.tif]

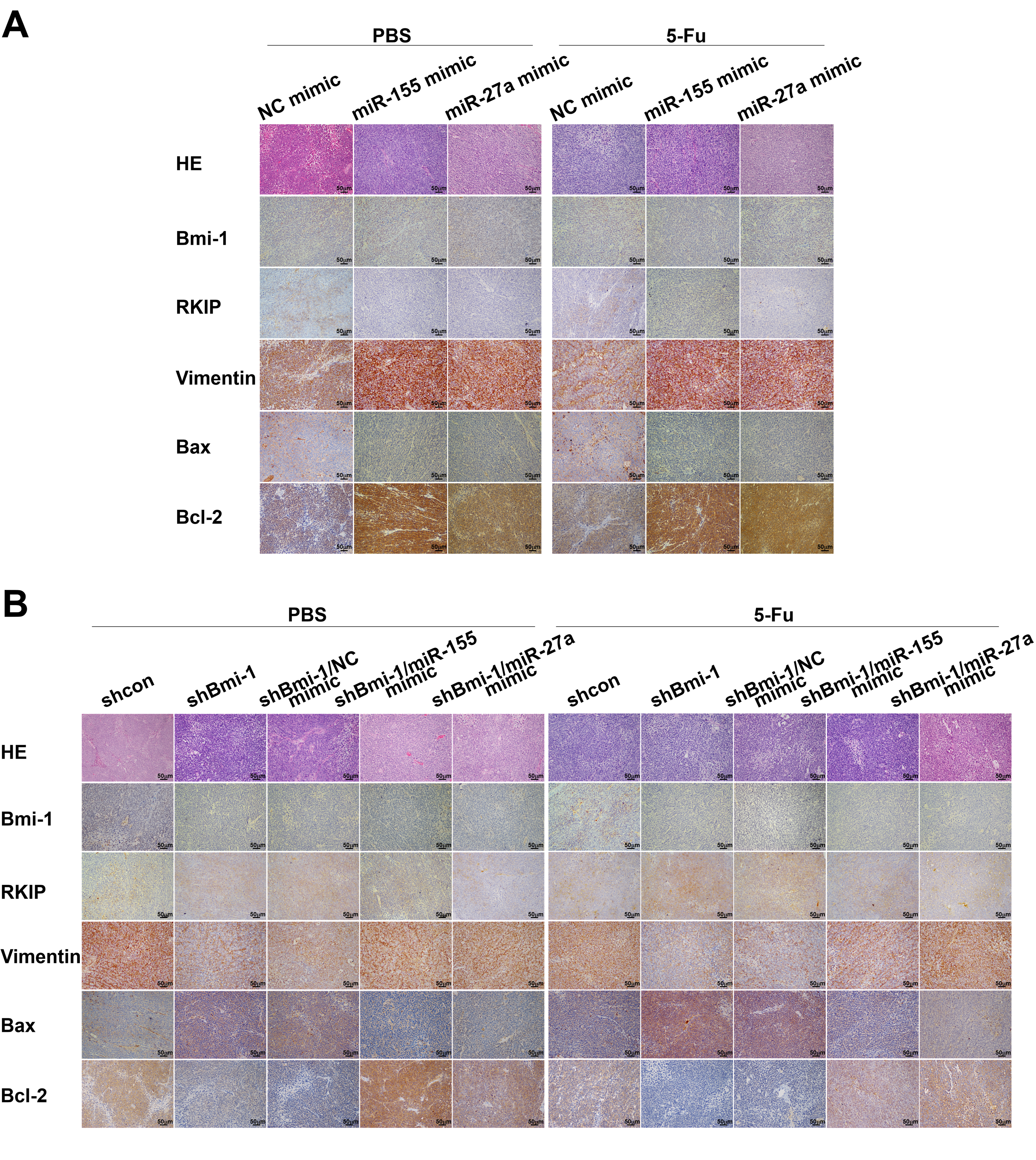

Supplement: Supplementary file 1 — Additional file 1: Fig. S1 The association between clinical data and Bmi-1 and RKIP. A. qRT-PCR analysis of Bmi-1 and RKIP RNA expression in 15 paired GC tissues (T) and adjacent normal tissue samples (N). B. Western blotting analysis of Bmi-1 and RKIP in 15 paired GC tissues. The definitions of T and N were the same as mentioned in A. C. Kaplan-Meier analysis of the 3-year overall survival of patients with intestinal-type or diffuse-type GC from TCGA. D. Bmi-1, miR-27a and miR-155 were upregulated, while RKIP was downregulated significantly in GC tissues from the TCGA database. *P < 0.05, **P < 0.01. Fig. S2 Bmi-1 does not upregulate RKIP at the mRNA level nor induce RKIP protein degradation. A. Bmi-1 and RKIP mRNA expression in GES-1 cells overexpressing Bmi-1. *P < 0.05 vs. GES-1-Vector. B. GES-1-Bmi-1 cells and GES-1-Vector cells were subjected to the protein synthesis inhibitor cycloheximide for the indicated period of time. The half-life of RKIP protein in Bmi-1-transduced cells was comparable to that in the control cells, which indicated that Bmi-1 did not induce RKIP protein degradation. Fig. S3 Quantification of Western blotting assays as well as invasion and migration assays. A. The densitometry analysis of bands from the Western blotting assays in Fig. 2f. *P < 0.05 vs. NC mimic/NC inhibitor. B. The densitometry analysis of bands from the Western blotting assays in Fig. 3a. *P < 0.05 vs. Vector-Ctrl/siNC. C. Analysis of the quantities of invading cells in migration and invasion assays. *P < 0.05 vs. shcon, **P < 0.01 vs. shcon/Vector-Ctrl, ##P < 0.01 vs. NC mimic/NC inhibitor. Fig. S4 miR-27a inhibitor and miR-155 inhibitor weakened the effects of Bmi-1 overexpression in functional experiments. A. Bmi-1 upregulation induced gastric cancer cell migration and invasion, which were decreased by the miR-155 inhibitor or miR-27a inhibitor (100 × magnification). B. The reduced ability of cell proliferation due to the transient transfection of the miR-155 inhibit [file 12943_2020_1229_MOESM1_ESM.zip › Figure S5.tif]
